# All‐Ferroelectric Spiking Neural Networks via Morphotropic Phase Boundary Neurons

**DOI:** 10.1002/advs.202407870

**Published:** 2024-10-09

**Authors:** Jangsaeng Kim, Eun Chan Park, Wonjun Shin, Ryun‐Han Koo, Jiseong Im, Chang‐Hyeon Han, Jong‐Ho Lee, Daewoong Kwon

**Affiliations:** ^1^ Department of Electronic Engineering Hanyang University Seoul 04763 Republic of Korea; ^2^ Department of Electrical and Computer Engineering and Inter‐university Semiconductor Research Center Seoul National University Seoul 08826 Republic of Korea; ^3^ Department of Semiconductor Convergence Engineering Sungkyunkwan University Suwon 16419 Republic of Korea; ^4^ Ministry of Science and ICT Sejong 30121 Republic of Korea

**Keywords:** double‐gate, leaky integrate‐and‐fire neuron, morphotropic phase boundary, neuromorphic, spike‐frequency adaptation, spiking neural networks

## Abstract

Artificial neurons and synapses are crucial for efficiently implementing spiking neural networks (SNNs) in hardware. The distinct functional requirements of artificial neurons and synapses present significant challenges in the implementation of area‐ and energy‐efficient SNNs. This study reports an all‐ferroelectric SNN system through co‐optimization of material properties and device configurations using wafer‐scale atomic layer deposition. For the first time, a double‐gate (DG) morphotropic phase boundary‐based thin‐film transistor (MPBTFT) is utilized for a leaky integrate‐and‐fire (LIF) neuron. The DG MPBTFT‐based LIF neuron eliminates the need for capacitors and reset circuits, thereby enhancing area and energy efficiency. The DG configuration demonstrates various neuronal functions with high reliability. Co‐optimizing materials and devices significantly enhance the performance and functional versatility of artificial neurons and synapses. Meticulous material engineering facilitates the seamless co‐integration of DG MPBTFT‐based neurons, ferroelectric thin‐film transistor (TFT)–based synapses, and normal TFTs on a single wafer. All‐ferroelectric SNN systems achieved a high classification accuracy of 94.9%, thereby highlighting the potential of DG MPBTFT‐based LIF neurons for advanced neuromorphic computing.

## Graphical abstract

This study reports an all‐ferroelectric spiking neural network system through co‐optimization of material properties and device configurations using wafer‐scale atomic layer deposition. Co‐optimizing materials and devices significantly enhance the performance and functional versatility of artificial neurons and synapses. Meticulous material engineering facilitates the seamless co‐integration of neurons, synapses, and normal thin‐film transistors on a single wafer.

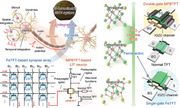

## 1 Introduction

**Figure 1:**
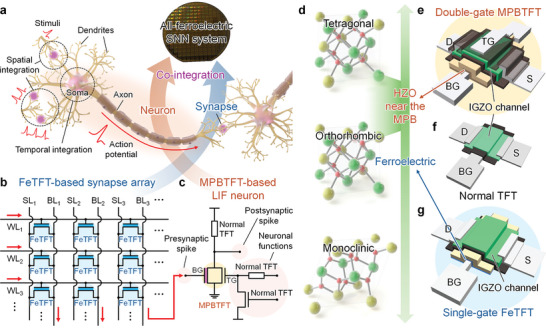
All‐ferroelectric spiking neural network (SNN) system co‐integrating artificial neurons and synapses. a) Schematic of biological neurons and synapses. Biological neurons comprise three main components: dendrites, soma, and axon. Neuronal dendrites receive stimuli via synapses from presynaptic neurons. These stimuli are spatiotemporally integrated into the soma and generate an action potential when the membrane potential exceeds a certain threshold. The generated action potential is transmitted through the axon to the synapses, reaching the postsynaptic neuron. b) Artificial synapse array based on nonvolatile ferroelectric thin‐film transistors (FeTFTs). c) Artificial neuron based on a volatile morphotropic phase boundary‐based thin‐film transistor (MPBTFT). A double‐gate (DG) configuration with four terminals enables effective neuronal functions: spike‐frequency adaptation (SFA) and lateral inhibition functions. d) Material optimization for hafnium zirconium oxide (HZO) near the morphotropic phase boundary (MPB). The MPB between the o‐phase and t‐phase can be obtained by precisely controlling the Zr content in the HZO thin film. The inherent partial polarization switching and volatile memory characteristics of HZO near the MPB are suitable for artificial neurons because they reduce the hardware burden by eliminating the need for membrane capacitors and complex reset circuits. The nonvolatile ferroelectric properties of HZO are suitable for artificial synapses. This approach enhances the area and energy efficiency of artificial neurons and synapses by co‐optimizing material properties and device configurations. Seamless co‐integration of e) DG MPBTFT‐based neurons, f) normal indium–gallium–zinc oxide (IGZO) thin‐film transistors (TFTs), g) and FeTFT‐based synapses on a single wafer facilitates implementation of all‐ferroelectric SNN system.

The advent of deep neural networks has marked a significant milestone in computational technology.^[^ ^1^, ^2^, ^3^, ^4^, ^5^, ^6^ ^]^ However, these systems often rely on the conventional von Neumann computing architecture, which suffers from critical limitations in area and energy efficiency.^[^ ^7^, ^8^, ^9^, ^10^, ^11^, ^12^ ^]^ Neuromorphic computing architecture inspired by the biological mechanisms of the human nervous system has been explored as a potential approach for realizing more efficient computing paradigms.^[^ ^13^, ^14^, ^15^ ^]^ Spiking neural networks (SNNs), which have garnered significant interest for their high biological similarity to the human brain, offer numerous advantages, including low power consumption, rapid inference capabilities, and event‐driven information processing.^[^ ^16^ ^]^ Central to advancing neuromorphic computing for SNNs is the efficient implementation and co‐integration of artificial neurons and synapses (**Figure** **1**a).^[^ ^17^, ^18^ ^]^ Beyond the individual implementation of artificial neurons and synapses, which are crucial components of SNNs, the seamless co‐integration of these components is essential for the development of area‐ and energy‐efficient SNNs.

Compared to extensive research on enhancing synaptic functionality using various material systems, the optimization and co‐integration of neuron functionality have been significantly underexplored. Many existing hardware demonstrations of neurons leave a significant gap between the current implementations and the desired performance for large‐scale robust neuromorphic systems, such as minimizing hardware complexity, enhancing energy efficiency, and ensuring reliable operation.^[^ ^18^, ^19^, ^20^, ^21^, ^22^, ^23^ ^]^ Early studies that attempted to emulate biological neurons employed complementary metal‐oxide‐semiconductor (CMOS) circuits.^[^ ^24^, ^25^, ^26^, ^27^, ^28^ ^]^ However, the intricate design of CMOS circuits and their limited biological similarity presented significant obstacles in achieving the area and energy efficiency.^[^ ^24^, ^25^, ^26^, ^27^, ^28^, ^29^ ^]^ Various emerging devices, including memristors,^[^ ^30^, ^31^, ^32^, ^33^, ^34^ ^]^ phase‐change memory,^[^ ^35^, ^36^ ^]^ magnetic tunnel junctions,^[^ ^37^, ^38^ ^]^ and insulator‐metal‐transition oscillators,^[^ ^39^, ^40^ ^]^ have been reported to overcome these limitations. Although these emerging devices emulate neuronal functions, their practical application in large‐scale neuromorphic systems remains constrained because of the need for additional capacitors and the lack of practical co‐integration methods.^[^ ^30^, ^31^, ^32^, ^33^, ^41^ ^]^ In particular, memristive neurons, the most promising candidates for hardware cost reduction, encounter challenges such as high electroforming voltage and limited reproducibility attributable to temporal and spatial variations.^[^ ^42^, ^43^ ^]^

The discovery of ferroelectricity in hafnium oxide (HfO_2_)‐based thin films, originating from the orthorhombic (o)‐phase, presented a promising solution to these challenges.^[^ ^44^, ^45^, ^46^, ^47^, ^48^, ^49^ ^]^ The gradual polarization switching in hafnium zirconium oxide (HZO) thin films demonstrated the implementation of capacitor‐less artificial neurons and synapses with excellent energy efficiency.^[^ ^50^, ^51^, ^52^, ^53^, ^54^ ^]^ While the nonvolatile memory characteristics of ferroelectric devices are well‐suited for artificial synapses, they do not align with the requirements for artificial neurons. Artificial neurons based on ferroelectric devices often necessitate complex reset circuits and feedback paths or specialized designs to reset after each firing event, which leads to increased hardware costs, energy consumption, and system complexity.^[^ ^49^, ^50^, ^51^, ^52^ ^]^ To address these issues, anti‐ferroelectric materials with volatile memory characteristics have been utilized for artificial neurons.^[^ ^20^, ^55^, ^56^ ^]^ Recent advances in material engineering introduced HZO near the morphotropic phase boundary (MPB) between the ferroelectric o‐phase and the anti‐ferroelectric tetragonal (t)‐phase.^[^ ^57^, ^58^ ^]^ Thus far, HZO thin films near the MPB were primarily utilized in dynamic random‐access memory (DRAM), which requires high capacitor performance because of their high dielectric constants.^[^ ^58^, ^59^ ^]^ However, their volatile memory characteristics and greater polarization at voltages lower than those of anti‐ferroelectrics render them ideal candidates for implementing energy‐efficient leaky integrate‐and‐fire (LIF) neurons. MPB‐based thin‐film transistors (MPBTFTs) can implement capacitor‐less neurons with low power consumption, high reliability, endurance, and scalability (detailed in Note S1, Supporting Information).

Implementing specific neural mechanisms essential for high performance, such as spike‐frequency adaptation (SFA) and lateral inhibition, is another challenge when developing area‐ and energy‐efficient SNNs. An SFA is a biological process that modulates neuronal activity during prolonged stimuli, preventing hyperactivity while maintaining network stability and responsiveness to new stimuli.^[^ ^60^ ^]^ Although incorporating SFA in artificial neurons significantly enhances the functionality and efficiency of SNNs, the complex CMOS circuitry introduces significant hardware overhead.^[^ ^50^, ^61^ ^]^ These constraints compromise the area and energy efficiency of artificial neurons, thereby highlighting the importance of optimizing device configuration. Lastly, neurons that meet all of these criteria must be seamlessly integrated with the synapses and transistors that constitute the circuit at a wafer scale. This implementation poses significant challenges, particularly in epitaxy‐based hafnium ferroelectric systems.^[^ ^62^, ^63^ ^]^

To address these issues, in this study, we present a complete all‐ferroelectric SNN system through seamless co‐integration of nonvolatile ferroelectric thin‐film transistors (FeTFTs) (Figure 1b), volatile MPBTFTs (Figure 1c), and normal indium–gallium–zinc oxide (IGZO) thin‐film transistors (TFTs) using a wafer‐scale atomic layer deposition (ALD) technique. A novel double‐gate (DG) MPBTFT‐based LIF neuron is demonstrated by optimizing material and device configuration (Figure 1d,e). The inherent partial polarization switching and volatile memory characteristics of HZO near the MPB obviate the need for additional capacitors and complex reset circuits in artificial neurons. By leveraging a DG configuration with four terminals, the DG MPBTFT‐based LIF neuron processes input pulses via the bottom‐gate (BG), while the top‐gate (TG) manages the SFA and lateral inhibition functions (Figure 1c). This enables the area‐ and energy‐efficient modulation of the firing frequency by adjusting the TG bias. Co‐optimization of materials and devices further enhances the area and energy efficiency of artificial neurons and synapses. Meticulous engineering of HZO (Figure 1d) facilitates the seamless co‐integration of DG MPBTFT‐based neurons, FeTFT‐based synapses, and normal IGZO TFTs on a single wafer while enhancing their functional versatility (Figure 1e–g). The performance of the all‐ferroelectric SNN system leveraging DG MPBTFT‐based LIF neurons and FeTFT‐based synapses was evaluated using the MNIST dataset. The SNN exhibits consistent performance for device variations with low energy consumption, thereby demonstrating the potential of DG MPBTFT‐based LIF neurons for advanced neuromorphic computing systems.

## 2 Results and Discussion

### 2.1 Biological Neuronal Functions

**Figure 2:**
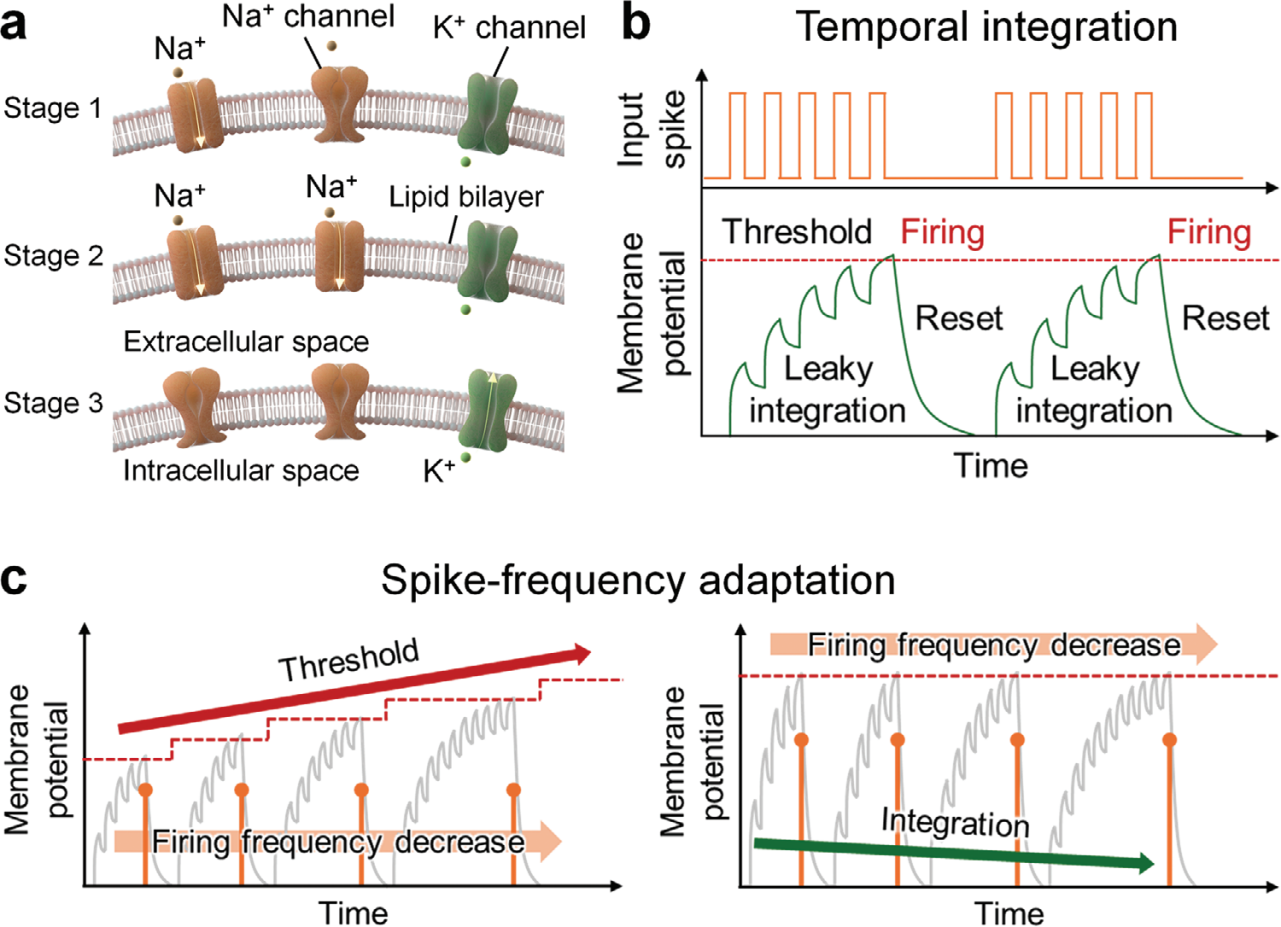
Biological neuronal functions. a Integrate‐and‐fire dynamics of biological neurons involving a series of ionic channel activities. b) Membrane potential integration and reset behaviors of neurons. When the membrane potential of a neuron exceeds a certain threshold, it triggers the generation of an action potential. Subsequently, the membrane potential resets to its initial state to prepare for processing the next input signals. c) Two primary methods to implement the SFA function. The left panel illustrates the modulation of neuronal firing frequency by increasing the threshold. In contrast, the right panel shows modulation by attenuating the neuronal integration function. DG MPBTFT‐based artificial neurons implement the SFA function by modulating the integration function.

Biological neurons spatially and temporally integrate stimuli from presynaptic neurons into the soma via neuronal dendrites (Figure 1a). When the membrane potential of a neuron surpasses a certain threshold, an action potential is generated and transmitted to a postsynaptic neuron via an axon,^[^ ^18^ ^]^ which involves a series of ionic channel activities (**Figure** **2**a). When stimuli are transmitted to the soma through the dendrites, a few Na^+^ channels are activated, allowing Na^+^ ions to flow into the intracellular space and gradually increase the membrane potential (Stage 1). As more stimuli are received, additional Na^+^ channels are activated, resulting in a significant influx of Na^+^ ions and a rapid rise in membrane potential (Stage 2). Once the membrane potential exceeds a certain threshold, Na^+^ channels are deactivated, and K^+^ channels are activated (Stage 3). This activation allows K^+^ ions to flow into the extracellular space, which decreases the membrane potential. The membrane potential integration and reset behaviors of the neurons are presented in Figure 2b. Figure 2c shows two primary methods for implementing the SFA function in LIF neurons: modulation of the threshold for neuronal firing (left panel) and modulation of neuronal integration function (right panel). DG MPBTFT‐based LIF neurons modulate the neuronal integration function for effective SFA.

### 2.2 Materials and Electrical Characteristics of DG MPBTFT

**Figure 3:**
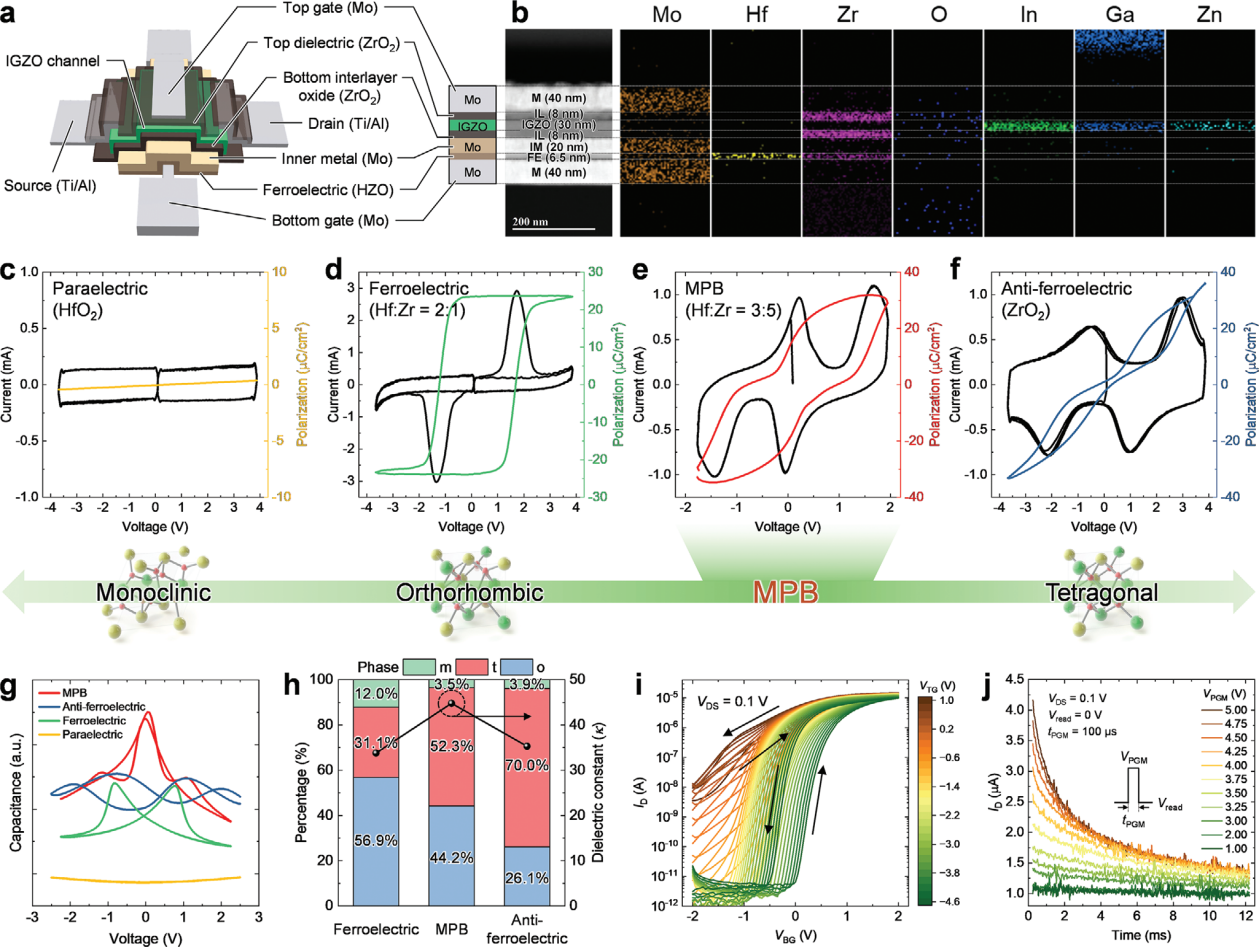
Materials engineering for HZO near the MPB of DG MPBTFT. a) Schematic and cross‐sectional view of DG MPBTFT. The device incorporates an MFMIS structure on the BG side and an MIS structure on the TG side, with both configurations sharing an IGZO channel. b) Cross‐sectional TEM image and EDS analysis of the fabricated DG MPBTFT. Switching current and *P*–*V* hysteresis loop through the PUND measurements with 100 kHz triangular pulses for c paraelectric, d) ferroelectric, e) MPB, and f) anti‐ferroelectric materials. The phase transition in HZO evolves from the m‐phase to the o‐phase and then to the t‐phase with an increase in the Zr content. This transition results in a range of properties, from paraelectric to ferroelectric and then to anti‐ferroelectric. Notably, the HZO thin film near the MPB, which exists between ferroelectric and anti‐ferroelectric properties, demonstrates a high polarization at relatively low voltages compared to anti‐ferroelectric materials. This feature is advantageous for low‐power neuronal operations. g) Capacitance of HZO with various Zr composition ratios. The HZO near the MPB exhibits the highest capacitance near 0 V. h) Relative phase ratios and dielectric constants for different material types. Ferroelectric materials predominantly exhibit the o‐phase, whereas the t‐phase characterizes anti‐ferroelectric materials. HZO near the MPB exhibits the highest dielectric constant, which is beneficial for reducing equivalent oxide thickness (EOT). i) Hysteretic transfer characteristics (*I*_D_–*V*_BG_) of DG MPBTFT under various TG voltage (*V*_TG_) conditions. A counterclockwise hysteresis loop is observed during the bidirectional direct current (DC) sweeps of the BG voltage (*V*_BG_) in the range of –2.0 V to 2.0 V. The measurements were conducted at a *V*_DS_ of 0.1 V. j) Volatile memory characteristics of DG MPBTFT. The *I*_D_ of the DG MPBTFT decays over time following the application of a 100 µs PGM pulse with various amplitudes. The *I*_D_ returns to its initial state once the applied pulse is removed, demonstrating the spontaneous reset function of LIF neurons.

**Figure** **3**a presents a schematic and cross‐sectional view of a DG MPBTFT, featuring a metal–ferroelectric–metal–insulator–semiconductor (MFMIS) structure on the BG side and a metal–insulator–semiconductor (MIS) structure on the TG side, both sharing an IGZO channel. The polarization of the MPB layer is modulated by both voltages applied to the BG and TG, thereby enabling the versatile and precise control of the device. Cross‐sectional transmission electron microscopy (TEM) image and energy‐dispersive spectroscopy (EDS) analysis verified the stacked structure of the fabricated DG MPBTFT (Figure 3b). The fabrication process for the DG MPBTFT is presented in the Experimental Section and Figure S1 (Supporting Information). The top optical images of the DG MPBTFT array are shown in Figure S2 (Supporting Information).

The phase transition in HZO progresses from the monoclinic (m)‐phase to the o‐phase and then to the t‐phase with an increasing Zr content, thereby exhibiting a spectrum of properties from paraelectric to ferroelectric to anti‐ferroelectric.^[^ ^57^, ^58^ ^]^ The DG MPBTFT leverages the HZO thin film near the MPB, which exists between ferroelectric and anti‐ferroelectric properties, for effective neuronal operation. The switching current and polarization relative to the applied voltage were obtained using positive‐up‐negative‐down (PUND) measurements in metal–ferroelectric–metal (MFM) capacitors incorporating HZO with varying Zr composition ratios (Figure 3c–f). Hafnium oxide (HfO_2_), which does not contain Zr, is dominated by the m‐phase and exhibits paraelectric properties (Figure 3c). Paraelectric materials are relatively less affected by electric fields than other materials. The formation of the o‐phase becomes dominant in HZO (Hf:Zr = 2:1), which contains both Hf and Zr (Figure 3d). This structure exhibits nonvolatile memory characteristics that retain polarity even after removing the applied electric field. The ferroelectric properties of this structure originate from the movement of the oxygen ions bonded to three metal atoms.^[^ ^58^ ^]^ The t‐phase is promoted with an increase in the concentration of Zr in HZO. Eventually, pure zirconium oxide (ZrO_2_) is dominated by the t‐phase and exhibits anti‐ferroelectric properties (Figure 3f); this structure exhibits volatile memory characteristics, in which the polarity induced by the electric field mostly disappears near 0 V. It is important to note that by meticulously engineering the Zr composition ratio in HZO (Hf:Zr = 3:5), an MPB between the ferroelectric and anti‐ferroelectric states can be obtained (Figure 3e; Note S2, Supporting Information). Near this MPB, HZO can achieve high polarization at relatively lower voltages than anti‐ferroelectric materials, facilitating energy‐efficient neuronal operation. Furthermore, the volatile memory characteristics of MPB are suitable for emulating the spontaneous leaky effects of LIF neurons.

Figure 3g shows the capacitance‐voltage relationships of HZO thin films with various Zr composition ratios. Ferroelectric and anti‐ferroelectric materials feature butterfly‐ and ribbon‐shaped curves, respectively, whereas paraelectric materials are relatively less affected by electric fields. HZO near the MPB demonstrates the highest capacitance near 0 V. X‐ray diffraction (XRD) analyses were conducted to investigate the crystallinity of HZO with various Zr contents (Figure S3, Supporting Information). Relative phase ratios derived from XRD and the dielectric constants for each material type are shown in Figure 3h. In all three material types, the non‐ferroelectric m‐phase is minimally present. Ferroelectric materials predominantly exhibit the o‐phase, whereas the t‐phase characterizes anti‐ferroelectric materials. The HZO near the MPB exhibits the highest dielectric constant, which is advantageous for reducing the equivalent oxide thickness (EOT).

Figure 3i shows the hysteretic transfer characteristics of DG MPBTFT under various TG voltage (*V*_TG_) conditions, which exhibit a counterclockwise hysteresis loop. Note that the electrical characteristics of DG MPBTFT with an MFMIS structure can be enhanced by adjusting the area ratio (AR) between the MFM (*A*_FE_) and MIS layers (*A*_MOS_).^[^ ^64^, ^65^ ^]^ The AR (*A*_FE_/*A*_MOS_) between the *A*_FE_ and *A*_MOS_ determines the capacitance ratio between the MFM and MIS layers within the MFMIS structure. Decreasing the capacitance of the MFM layer while maintaining the capacitance of the MIS layer intensifies the electric field across the MFM layer. This intensification in the electric field enhances the polarization within the MFM layer, thereby improving the memory window of the device. The proper capacitance matching between these layers mitigates the electric field across the insulating layer, substantially enhancing the reliability of the device.^[^ ^64^ ^]^ The transfer characteristics of the DG MPBTFT for various ARs are shown in Figure S4 (Supporting Information). Furthermore, the inner metal layer within the MFMIS structure rectifies the inhomogeneous polarization in the ferroelectric layer, ensuring uniform electric field distribution and channel conductivity. This results in reliable device operations with reduced cycle‐to‐cycle variation of the device.

The volatile memory characteristics of the DG MPBTFT were investigated by applying a single program (PGM) pulse with various amplitudes (*V*_PGM_s) and a fixed pulse width (*t*_PGM_) of 100 µs. Following the PGM operation, a read operation was conducted at a constant voltage (*V*_read_) of 0 V. Figure 3j illustrates the drain current (*I*_D_) of the DG MPBTFT decaying over time after applying a PGM pulse. *V*_PGM_s from 1.0 to 5.0 V were applied at the same initial state; higher *V*_PGM_s resulted in a greater increase in *I*_D_, which then decayed more rapidly at the outset upon removing the PGM pulse. This behavior, in which *I*_D_ returns to its initial state once the applied pulse is removed, effectively mirrors the spontaneous reset characteristics essential for LIF neurons. Figure S5 (Supporting Information) presents the *I*_D_ responses of DG MPBTFT to successive PGM pulses with various *V*_PGM_s.

### 2.3 Neuronal Behavior of DG MPBTFT

**Figure 4:**
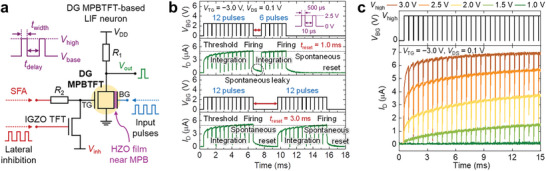
DG MPBTFT‐based LIF neuron. a) Schematic of a DG MPBTFT‐based LIF neuron. LIF neuron comprises a DG MPBTFT, normal IGZO TFT, and two resistors. Note that both resistors can be implemented using normal IGZO TFTs with an optimized resistance by modulating their widths and lengths. Normal IGZO TFTs and DG MPBTFTs are co‐integrated on a single wafer. Leveraging the inherent partial polarization switching and polarization volatility of DG MPBTFT eliminates the necessity of capacitors and complex reset circuits, thereby enhancing area and energy efficiency. While the BG processes input pulses, the TG manages SFA and lateral inhibition functions. The inset shows the input pulse train with four variables: voltage levels (*V*_high_ and *V*_base_), pulse width (*t*_width_), and interval between pulses (*t*_delay_). b) Neuronal behavior of DG MPBTFT with the application of consecutive input pulses to the BG. The polarization of HZO gradually switches in response to the applied input pulses and exhibits a spontaneous leaky effect caused by polarization volatility. DG MPBTFT demonstrates the capability for emulating both integrate‐and‐fire and spontaneous reset functions of biological neurons without capacitors and external reset circuitry. After firing and resetting, the LIF neuron can fire again in response to subsequent input pulse trains. c) *I*_D_ response of DG MPBTFT to consecutive input pulse trains with various *V*_high_s. Increasing *V*_high_ enhances the *I*_D_ change and strengthens the integration function of the LIF neurons. In contrast, excessively low *V*_high_ impairs the integration function of LIF neurons, thereby underscoring the necessity of an optimal *V*_high_.

The inherent partial polarization switching and polarization volatility of DG MPBTFTs effectively emulate the integration and spontaneous reset functions of biological neurons, respectively, thereby facilitating the implementation of artificial LIF neurons. **Figure** **4**a shows a schematic of a DG MPBTFT‐based LIF neuron, which consists of a DG MPBTFT, normal IGZO TFT, and two resistors (*R*_1_ and *R*_2_). Leveraging the partial polarization switching of HZO eliminates the need for capacitors, which occupy large areas in artificial neurons. The output voltage (*V*_out_), expressed as the resistance ratio between *R*_1_ and DG MPBTFT, can be adjusted by modulating the resistance of *R*_1_ connected to the drain of the DG MPBTFT. Resistance of *R*_2_ connected to the TG of the DG MPBTFT is higher than that of the on‐state normal IGZO TFT but lower than that of the off‐state normal IGZO TFT. It is worth noting that both resistors can be implemented using normal IGZO TFTs with an optimized resistance by modulating their widths and lengths. Normal IGZO TFTs and DG MPBTFTs were co‐integrated on a single wafer, as shown in Figure S1 (Supporting Information). This co‐integration enhances area and process efficiency, offering significant advantages for advanced neuromorphic systems. The transfer characteristics of the fabricated normal IGZO TFT are depicted in Figure S6 (Supporting Information).

Input pulses applied to the BG of DG MPBTFT partially switch the polarity of HZO to implement the integration function of the LIF neurons. On the other hand, the TG of the DG MPBTFT serves two roles: SFA and lateral inhibition. The SFA controls the firing frequency of LIF neurons by modulating the *V*_TG_ in the DG MPBTFT. The effect of *V*_TG_ on operating the DG MPBTFT is verified in Figure 3i. This function is essential for enhancing the performance of SNNs by preventing the repetitive firing of specific neurons and ensuring balanced neuronal activity.^[^ ^50^, ^52^, ^60^, ^66^ ^]^ In addition, lateral inhibition is achieved by applying an inhibitory pulse to the gate of a normal IGZO TFT upon activating other neurons. Consequently, an inhibitory voltage (*V*_inh_) is applied to the TG of the DG MPBTFT, effectively inhibiting the LIF neuronal firing. This mechanism plays a pivotal role in achieving high performance in SNNs.^[^ ^51^, ^67^, ^68^ ^]^ This section investigates the neuronal behavior in response to input pulses applied to the BG of the DG MPBTFT. The effects of varying voltage levels (*V*_high_ and *V*_base_), pulse width (*t*_width_), and the interval between pulses (*t*_delay_) within the input pulse train (inset in Figure 4a) on the neuronal behavior of DG MPBTFT are thoroughly analyzed.

Figure 4b shows the neuronal behavior of DG MPBTFT when consecutive input pulses are applied to the BG. An input pulse train with an amplitude, width, and interval between pulses of 2.5 V, 500 µs, and 10 µs, respectively, is applied (top panel). The polarization of HZO gradually switches in response to the applied input pulses, thereby effectively emulating the integration function of a neuron. Upon receiving 12 input pulses, the *I*_D_ of DG MPBTFT exceeds the threshold current (5.0 µA), thereby triggering the firing of the LIF neuron (second panel from the top). After firing, the LIF neurons exhibit a spontaneous leaky effect attributed to the polarization volatility of HZO. The subsequent application of the input pulse train before the complete reset of the LIF neuron (reset time = 1.0 ms) results in neuronal firing after 6 pulses, fewer than initially required for firing. The neuron reverts to its initial state through a spontaneous reset given sufficient time after firing. Increasing the reset time to 3.0 ms (third panel from the top) ensures a complete reset of the LIF neuron. The neuron fires again in response to the 12 input pulses (bottom panel). These behaviors highlight the capability of DG MPBTFT to mimic both the integrate‐and‐fire and spontaneous reset functions of biological neurons, obviating the need for external reset circuitry.

The amplitude of the pulses applied to the BG of the DG MPBTFT directly affects the extent of polarization switching in the HZO. Figure 4c depicts the *I*_D_ response of the DG MPBTFT to consecutive input pulse trains with various *V*_high_s, while maintaining *t*_width_ of 500 µs and *t*_delay_ of 10 µs. For most *V*_high_s, the *I*_D_ increases following pulse application. In contrast, at a *V*_high_ of 1.0 V, which is insufficient to switch the polarization of HZO, there is no change in *I*_D_ regardless of the pulse application. The polarization of HZO switches more extensively in response to each pulse with an increase in *V*_high_, thereby augmenting the *I*_D_ change and strengthening the integration function of the LIF neurons. However, excessively high *V*_high_s overly enhance the integration function, enabling the neuron to fire with a single input pulse. This condition compromises the resolution of input pulse detection in the LIF neuron and subsequently degrades network performance. Conversely, excessively low *V*_high_s impair the integration function of neurons, highlighting the importance of selecting an appropriate *V*_high_.

**Figure 5:**
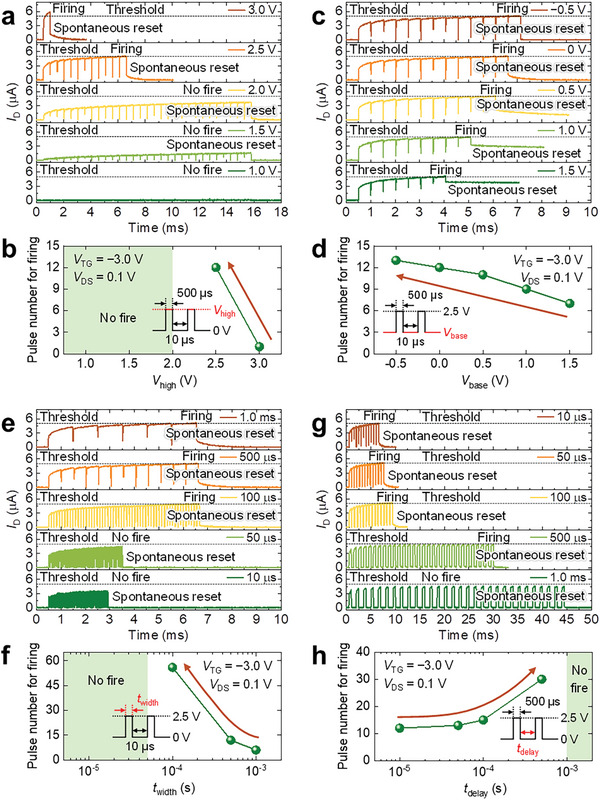
Neuronal behavior modulation of DG MPBTFT with various input pulse conditions. a) Neuronal behavior of DG MPBTFT under various *V*_high_ conditions with fixed *V*_base_, *t*_width_, and *t*_delay_ of 0 V, 500 µs, and 10 µs, respectively. A greater number of pulses is required for neuronal firing with a decrease in *V*_high_, and the neuron fails to fire below a certain *V*_high_ condition. b) Number of pulses required to trigger neuronal firing as a function of *V*_high_. c) Neuronal behavior of DG MPBTFT under various *V*_base_ conditions with fixed *V*_high_, *t*_width_, and *t*_delay_ of 2.5 V, 500 µs, and 10 µs, respectively. A low *V*_base_ accelerates the polarization reversion to its initial state, strengthening the leaky effect. Consequently, a greater number of pulses is required for neuronal firing with a decrease in *V*_base_. d) Number of pulses required to trigger neuronal firing as a function of *V*_base_. e) Neuronal behavior of DG MPBTFT under various *t*_width_ conditions with a fixed *V*_high_, *V*_base_, and *t*_delay_ of 2.5 V, 0 V, and 10 µs, respectively. An extended *t*_width_ results in more extensive polarization switching in response to a single input pulse. A greater number of pulses is required for neuronal firing with a decrease in *t*_width_, and neuron fails to fire below a certain *t*_width_ condition. f) Number of pulses required to trigger neuronal firing as a function of *t*_width_. g) Neuronal behavior of DG MPBTFT under various *t*_delay_ conditions with a fixed *V*_high_, *V*_base_, and *t*_width_ of 2.5 V, 0 V, and 500 µs, respectively. The polarization induced by the input pulse is significantly lost during the interval between pulses with an increase in *t*_delay_, thereby requiring a greater number of pulses for neuronal firing. The neuron fails to fire beyond a certain *t*_delay_ condition. h) Number of pulses required to trigger neuronal firing as a function of *t*_delay_.

The neuronal behaviors of DG MPBTFT were further investigated under four input pulse variables: *V*_high_, *V*_base_, *t*_width_, and *t*_delay_. **Figure** **5**a,b shows neuronal behavior under various *V*_high_ conditions while maintaining a *V*_base_, *t*_width_, and *t*_delay_ of 0 V, 500 µs, and 10 µs, respectively. For a threshold current of 5 µA, a *V*_high_ of 3.0 V triggers neuronal firing with a single input pulse, whereas a *V*_high_ of 2.5 V requires 12 input pulses to elicit firing. For *V*_high_s below 2.0 V, *I*_D_ remains below the threshold current required for neuronal firing. After removing the input pulses, the neuron resets spontaneously to its initial state in all cases. The variation in the number of pulses required to trigger neuronal firing as a function of *V*_high_ is depicted in Figure 5b. An increasing number of pulses are required to trigger neuronal firing with a decrease in *V*_high_, and the neuron fails to fire below a certain *V*_high_ condition.

While the *V*_high_ of the input pulse affects the integration function of the DG MPBTFT‐based LIF neurons, *V*_base_, which represents the standby voltage applied when no input pulse is present, affects the spontaneous leaky effect of neurons. Figure S7 (Supporting Information) illustrates the spontaneous reset characteristics of neurons under various *V*_base_ conditions after applying 30 input pulses. A high *V*_base_ maintains the polarization of HZO and decelerates the spontaneous leaky effect, enhancing the integration function of the neurons. In contrast, a low *V*_base_ accelerates the polarization reversion of HZO to its initial state, strengthening the leaky effect of neurons. In addition, the polarization of HZO is sustained between the input pulses with a high *V*_base_, thereby resulting in a higher *I*_D_ even with the application of identical 30 input pulses, compared to a lower *V*_base_. Figure 5c,d show the neuronal behavior of DG MPBTFT under various *V*_base_ conditions while maintaining a *V*_high_, *t*_width_, and *t*_delay_ of 2.5 V, 500 µs, and 10 µs, respectively. A low *V*_base_ enhances the spontaneous leaky effect of neurons, necessitating more input pulses to trigger neuronal firing. Conversely, a higher *V*_base_ mitigates the leaky effect and bolsters the integration function, enabling neurons to fire with fewer input pulses. However, this higher *V*_base_ also results in a slower reset to the initial state because of the decreased leaky speed. Figure 5d illustrates the relationship between *V*_base_ and the number of pulses required to trigger neuronal firing. The number of pulses required for neuronal firing increases with a decrease in *V*_base_.

The effect of *t*_width_ on the neuronal behavior is shown in Figure 5e,f. It is obvious that an extended *t*_width_ leads to more extensive polarization switching of HZO in response to a single input pulse. In contrast, the number of input pulses required to trigger neuronal firing increases with a decrease in *t*_width_. The neuron ceases to fire when *t*_width_ is reduced below a certain threshold, and this is attributed to a substantial loss of the induced polarization of HZO during the interval between pulses. This effect becomes particularly pronounced at a *t*_width_ of 10 µs, which equals *t*_delay_, thereby stabilizing the *I*_D_ that does not exceed a certain threshold. Figure 5g,h depict the effect of *t*_delay_ on neuronal behavior while maintaining a *V*_high_, *V*_base_, and *t*_width_ of 2.5 V, 0 V, and 500 µs, respectively. The polarization of HZO induced by the input pulse is significantly lost during the interval between pulses with an increase in *t*_delay_, weakening the integration function of the neurons. The HZO becomes depolarized during the interval between pulses when *t*_delay_ is extended beyond a certain threshold, thereby preventing neuronal firing. Contrary to the results shown in Figure 5e,f, where neurons failed to fire when both *t*_width_ and *t*_delay_ were 10 µs, neurons fired even when *t*_width_ and *t*_delay_ were the same (500 µs) because of the sufficiently large *t*_width_. These results highlight the importance of carefully adjusting the input pulse conditions to ensure the effective operation of DG MPBTFT‐based LIF neurons.

Endurance measurements through neuronal operation and MFM structure were further conducted to validate the reliable operation of the DG MPBTFT‐based LIF neurons. The neurons demonstrate consistent neuronal operation across 10^10^ firing cycles with slight degradation, thereby exhibiting substantial robustness (Figure S8, Supporting Information). The endurance characteristics can be enhanced through the optimization of input pulse conditions. Figure S9a (Supporting Information) shows a low device‐to‐device variation of 7.51% obtained across six DG MPBTFTs. The six DG MPBTFT‐based LIF neurons demonstrate high uniformity based on 600 neuronal firing activities (Figure S9b, Supporting Information). This consistency underscores the reliability and uniformity of the operational performance of neurons. Figure S10 (Supporting Information) depicts the neuronal operations of the DG MPBTFT‐based LIF neurons under reduced *V*_DS_ and *t*_width_ conditions. The neurons demonstrate stable integration and reset functions at a *V*_DS_ and *t*_width_ of 0.03 V and 50 µs, respectively. Therefore, the DG MPBTFT‐based LIF neuron consumes ≈1.95 pJ per spike (Note S3, Supporting Information). The energy consumption of neurons can be further reduced by decreasing the amplitude and width of the input pulse, as well as the operating voltage of the DG MPBTFTs, provided these adjustments are within the range of stable neuronal operations.

### 2.4 Spike‐Frequency Adaptation and Lateral Inhibition of DG MPBTFT‐Based LIF Neurons

The DG configuration of MPBTFT facilitates the functional diversity of LIF neurons. The BG receives input pulses and performs the integration function, whereas the TG implements the SFA and lateral inhibition functions. There are two primary methods for implementing the SFA function in LIF neurons. The first method (left panel of Figure 2c) involves modulating the neuronal firing frequency by incrementally increasing the threshold for each neuronal firing event. The second method (right panel of Figure 2c) modulates the neuronal firing frequency by attenuating the integration function of the neurons for each neuronal firing event. In the case of the DG MPBTFT‐based LIF neurons, the SFA function is implemented by modulating the integration function, effectively adjusting the neuronal firing frequency.

**Figure 6:**
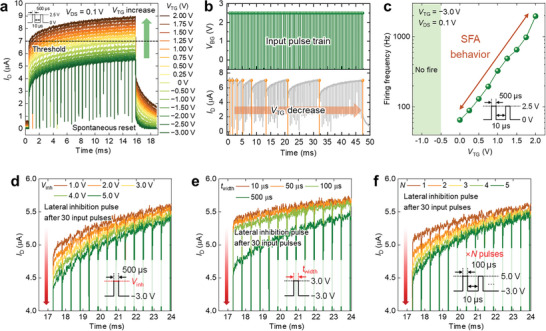
SFA and lateral inhibition functions of DG MPBTFT‐based LIF neuron. a) Neuronal behavior of DG MPBTFT under various *V*_TG_ conditions. Consistent input pulses are applied to the BG. The integration function of neurons can be modulated by adjusting the *V*_TG_. A high *V*_TG_ strengthens the integration function and reduces the required number of pulses for neuronal firing. b) Neuronal behavior of DG MPBTFT leveraging *V*_TG_ modulation when input pulses are applied at regular intervals. The reset time after neuronal firing is ignored for convenience. The gray and orange lines indicate the *I*_D_ response of DG MPBTFT and the firing event of the LIF neuron, respectively. *V*_TG_ decreases each time the neuron fires, weakening the integration function and requiring more input pulses for subsequent firings. The DG MPBTFT‐based LIF neuron demonstrates SFA behavior by utilizing the *V*_TG_ modulation. c) Firing frequency of DG MPBTFT‐based LIF neuron as a function of *V*_TG_. The SFA function is effectively implemented by adjusting the *V*_TG_. Lateral inhibition operations of DG MPBTFT‐based LIF neurons modulated by the d) *V*_inh_, e) *t*_width_, and f) *N* of inhibitory pulses. Insets show the inhibitory pulse conditions. The inhibitory operations were investigated following applications of 30 identical input pulses. The inhibitory efficiency is enhanced by increasing *V*_inh_, extending *t*_width_, and elevating *N*.

**Figure** **6**a shows the neuronal behavior of the DG MPBTFT under various *V*_TG_ conditions. A higher *V*_TG_ strengthens the integration function of neurons under consistent input pulse conditions, thereby reducing the number of input pulses required for neuronal firing and vice versa. This characteristic demonstrates that the firing frequency of DG MPBTFT‐based LIF neurons can be effectively modulated by adjusting *V*_TG_. Figure 6b shows the neuronal behavior of the DG MPBTFT leveraging *V*_TG_ modulation when input pulses are applied at regular intervals. *V*_TG_ decreases each time a neuron fires. A decrease in *V*_TG_ diminishes the integration function, necessitating more input pulses for the subsequent firing of the neuron. Consequently, modulating *V*_TG_ leads to a reduced firing frequency of neurons, manifesting as SFA behavior. Figure 6c depicts the firing frequency of the DG MPBTFT‐based LIF neurons as a function of *V*_TG_. For an identical input pulse train, the firing frequency of the neurons effectively adjusts in response to changes in *V*_TG_.

The pivotal role of TG extends to lateral inhibition, as depicted in Figure 4a. Figure 6d–f shows lateral inhibition operations of the DG MPBTFT‐based LIF neurons modulated by the voltage (*V*_inh_), width (*t*_width_), and number (*N*) of inhibitory pulses. These inhibition operations were investigated following the application of 30 identical input pulses. Inhibitory pulse conditions are shown in the insets. The polarization of HZO reverts to its initial state more rapidly with an increase in *V*_inh_, thereby enhancing the inhibitory efficiency, as demonstrated in Figure 6d. The subsequent application of the same input pulse train begins integration from a lower *I*_D_ when a high *V*_inh_ is utilized, thereby indicating effective inhibition. Likewise, an increase in *t*_width_ enhances the inhibitory efficiency, requiring more input pulses for subsequent neuronal firing (Figure 6e). The repeated application of inhibitory pulses progressively enhances their inhibitory effects on neuronal activity (Figure 6f). These operational characteristics confirm that DG MPBTFT‐based LIF neurons are capable of effectively performing lateral inhibition, a pivotal function in SNNs.

**Table** **1** presents a benchmarking analysis of the state‐of‐the‐art artificial neurons using emerging devices. Many previously reported artificial neurons necessitate membrane capacitors or reset circuits that occupy a large area. Other challenges also remain, such as high power consumption, difficulty in implementing SFA and inhibitory functions, and limited CMOS compatibility. Additionally, the majority of studies have focused on the characteristics of individual neurons, with only a few studies achieving the co‐integration of artificial neurons and synapses, presenting substantial challenges for implementing fully integrated neuromorphic systems. The proposed DG MPBTFT‐based LIF neuron effectively addresses these challenges through co‐optimization of material and device configuration. MPB, first used in artificial neurons, is CMOS compatible and eliminates the need for capacitors or reset circuits. The characteristic of exhibiting relatively high polarization at low voltage compared to anti‐ferroelectric materials enables low‐power operation. Furthermore, the DG configuration enables efficient implementation of SFA and inhibitory functions. The seamless co‐integration with synaptic FeTFTs facilitates a fully integrated neuromorphic system.

**Table 1:** Benchmarking analysis of the state‐of‐the‐art artificial neurons using emerging devices. Although various emerging devices have been utilized for artificial neurons, key challenges remain, such as low energy consumption, elimination of membrane capacitors and reset circuits, and incorporation of SFA and inhibitory functions. Moreover, while most studies have focused on the characteristics of individual neurons, only a limited number of studies demonstrated a fully integrated neuromorphic system with artificial synaptic devices. Our DG MPBTFT‐based LIF neuron is distinguished by its high endurance, low energy consumption, and capability to support SFA and inhibitory functions without the need for membrane capacitors and reset circuits. The energy consumption of LIF neurons can be minimized further by optimizing the input pulse conditions and operating voltage of the DG MPBTFTs within the range of stable neuronal operation. Furthermore, the SNN effectively integrates synaptic FeTFTs, DG MPBTFT‐based LIF neurons, and normal IGZO TFTs, demonstrating a seamless co‐integration of neuromorphic system components.

Devices	Operating voltage	Endurance	Energy consumption^a)^	Membrane capacitor	Reset mechanism	SFA function	Inhibitory function	CMOS compatibility	Co‐integration with synapses	
SiO_x_N_y_:Ag diffusive memristor	>1.0 V	>10^6^	≈60 pJ/spike^b)^	Yes (≈5 nF)	Spontaneous reset	Not available	Not available	Full	Yes	[30]
Ag/HfO_2_ memristor	≈0.5 V	–	≈20 pJ/spike	Yes (≈570 pF)	Spontaneous reset	Not available	Not available	Full	No	[32]
NbO_2_ memristor (IMT)	≈1.1 V	–	≈52 pJ/spike	Yes (≈380 pF)	Spontaneous reset	Not available	Not available	Partial	No	
VO_2_ memristor (IMT)	0.8 V	–	≈30 pJ/spike^b)^	Yes (1 nF)	Spontaneous reset	Not available	Not available	Partial	No	[33]
PCM (Ge_2_Sb_2_Te_5_)	1.5 V	3×10^9^	>50 pJ/spike	No	Reset circuit	Not available	Not available	Partial	No	[35]
MTJ (FeB/MgO/CoFeB)	1.0 V	–	7.1 fJ/spike (simulation)	No	Reset circuit	Not available	Not available	Partial	No	[38]
FeFET (PZT)	3.3 V	–	≈360 pJ/spike (simulation)	Yes (8 nF)	Reset circuit	Not available	Available	Partial	No	[49]
FeFET (HZO)	2.6 V	–	1–10 pJ/spike	No	Reset circuit	Available^c)^	Available	Full	Yes	[50]
Leaky‐FeFET (HZO)	1.8 V	–	≈420 pJ/spike^b)^	No	Spontaneous reset	Not available	Available	Full	No	[51]
AFeFET (HZO)	3.5 V	>10^9^	≈15 pJ/spike^b)^	No	Spontaneous reset	Not available	Available	Full	Yes	[55]
DG MPBTFT	2.5 V	>10^10^	≈1.95 pJ/spike	No	Spontaneous reset	Available	Available	Full	Yes	This study

IMT: insulator‐metal‐transition; PCM: phase‐change memory; MTJ: magnetic tunnel junction; FeFET: ferroelectric field‐effect transistor; PZT: lead zirconate titanate (PbZr_x_Ti_1‐x_O_3_); HZO: hafnium zirconium oxide (Hf_x_Zr_1‐x_O_2_); AFeFET: anti‐ferroelectric field‐effect transistor; DG: double‐gate; MPBTFT: morphotropic phase boundary‐based thin‐film transistor

^a)^ Energy consumption per spike;

^b)^ Calculated approximately from I−t and V−t curves;

^c)^ Implemented using three transistors and one capacitor.

### 2.5 Spiking Neural Networks with DG MPBTFT‐Based LIF Neurons

**Figure 7:**
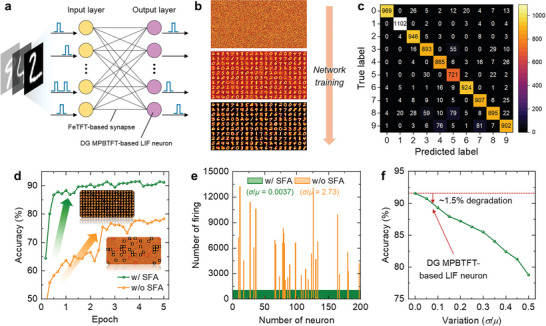
Spiking neural networks with DG MPBTFT‐based LIF neuron. a) All‐ferroelectric fully connected single‐layer SNN comprising FeTFT‐based synapses and DG MPBTFT‐based LIF neurons. b) Evolution of synaptic weight maps during network training. Randomly distributed initial synaptic weights progressively specialize for representing specific digits, thereby demonstrating effective network training. The diversity of digits represented within the trained weight map is closely related to the capability of SNNs for identifying handwritten digits. c) Confusion matrix obtained through SNN after network training. The SNN leveraging DG MPBTFT‐based LIF neurons successfully classifies ten types of handwritten digits. d) Classification accuracy of SNN with and without the SFA function. Insets show synaptic weight maps after network training. The SNN with the SFA function achieves a high classification accuracy because of sufficient updates across most synaptic weights. The synaptic weight map exhibits a diverse representation of digits. Conversely, the performance of the SNN without the SFA function is degraded because of the limited updates of synaptic weights associated with specific neurons. The synaptic weight map exhibits a reduced diversity of digit representation, and the effective engagement of numerous neurons is limited. e) Distribution of firing numbers of neurons with and without the SFA function. The SFA function promotes uniform firing across all neurons, enhancing network performance. In contrast, in the absence of SFA, firing is disproportionately concentrated in specific neurons, leaving others largely inactive. f) Classification accuracy of SNN as a function of the device‐to‐device variation of DG MPBTFTs. The SNN leveraging DG MPBTFT‐based LIF neurons demonstrates robust operation with a slight accuracy degradation of ≈1.5% because of a low device‐to‐device variation.

A handwritten digit recognition task was conducted to evaluate the performance of the DG MPBTFT‐based LIF neurons. An all‐ferroelectric fully connected single‐layer SNN consisting of FeTFT‐based synapses and DG MPBTFT‐based LIF neurons was utilized in the task (**Figure** **7**a). The configuration and operation of the SNN are described in detail in the Experimental Section. Similar fabrication processes of FeTFTs, DG MPBTFTs, and normal IGZO TFTs facilitate their co‐integration on a single wafer, as depicted in Figure S1 (Supporting Information). The electrical characteristics of the fabricated FeTFTs are detailed in Figure S11 (Supporting Information), which highlights their ability to emulate biological synapses because of their nonvolatile memory characteristics. The synaptic FeTFTs exhibit multilevel synaptic weights (6 bits) with highly linear weight update characteristics and low cycle‐to‐cycle variation of 9.19% over 20 cycles, ensuring their reliable operation. A detailed description of the weight update process for synaptic FeTFTs is provided in the Experimental Section.

The evolution of synaptic weight maps through network training is illustrated in Figure 7b. Although initial synaptic weights exhibit a random distribution, they represent specific digits as the training progresses, demonstrating effective network training. The diversity of the digits represented within the trained weight map directly affects the capability of SNNs to identify handwritten digits. After network training, the confusion matrix obtained through the SNN leveraging DG MPBTFT‐based LIF neurons validates the successful classification of ten types of handwritten digits (Figure 7c). Figure 7d shows the classification accuracy of the SNN with and without the SFA function. The insets represent the weight maps obtained after network training. Utilization of SFA results in sufficient updates across most synaptic weights and an abundant diversity of digit representations within the weight map. In contrast, as evidenced by the weight map, the absence of SFA leads to updates predominantly in the synaptic weights connected to specific neurons. This limitation curtails the effective utilization of numerous neurons and impedes the weight map from manifesting various types of digits, consequently degrading the network performance. Figure 7e depicts the distribution of the firing numbers of neurons with and without the SFA function. This result clarifies our preceding analyses, revealing distinct neuronal firing patterns contingent on the presence or absence of the SFA function. The presence of an SFA leads to uniform firing frequencies across all neurons, enhancing the network performance. In contrast, in the absence of SFA, specific neurons dominate the firing activity, leaving others largely inactive. These results highlight the pivotal role of the SFA function in efficiently utilizing LIF neurons. Figure 7f shows the classification accuracy of the SNN as a function of the device‐to‐device variation in DG MPBTFTs. Since DG MPBTFTs exhibit small device‐to‐device variations, SNN demonstrates robust operation with a slight accuracy degradation of ≈1.5%. This consistent performance is crucial for maintaining the reliability and effectiveness of neuromorphic systems. The performance of the SNN as a function of the cycle‐to‐cycle variation of FeTFT‐based synapses was further evaluated (Figure S12, Supporting Information). Stable operation with a slight accuracy degradation of ≈1.5% was verified owing to a low cycle‐to‐cycle variation of synaptic FeTFTs. Expanding the network size can enhance the performance of the SNN, achieving a classification accuracy of 94.9% with 2000 output neurons (Figure S13, Supporting Information). Increasing the number of neurons and synapses within the network can lead to more complex and capable neural architectures, potentially improving the network performance. Figure S14 (Supporting Information) shows the impacts of temperature on neuronal behavior and SNN performance. The performance of the SNN degrades as temperature increases.

## 3 Conclusion

In this study, we have demonstrated an all‐ferroelectric SNN system through seamless co‐integration of area‐ and energy‐efficient DG MPBTFT‐based LIF neurons and FeTFT‐based synapses. MPBTFT, which was first used to implement artificial neurons, effectively emulates the integration and spontaneous leaky/reset functions of LIF neurons by harnessing the inherent partial polarization switching and volatile memory characteristics. The DG MPBTFT‐based LIF neuron eliminates the need for capacitors or additional reset circuits, offering exceptional area and energy efficiency. Particularly, the co‐optimization of material and device configuration enables a low‐power LIF neuron with functional versatility. The HZO near the MPB achieves high polarization at relatively low voltages compared to anti‐ferroelectric materials, which is advantageous for low‐power neuronal operations. The DG configuration with four terminals allows the BG to process input pulses while the TG performs SFA and lateral inhibition functions. Adjusting the bias applied to the TG efficiently modulates the firing frequency of LIF neurons. The CMOS compatibility and similar structure of DG MPBTFTs, FeTFTs, and normal IGZO TFTs enable the seamless implementation of an all‐ferroelectric SNN system. The system achieved a high classification accuracy of 94.9% on the MNIST dataset with the successful implementation of the SFA function. The stable neuronal operations were maintained across 10^10^ firing cycles, ensuring excellent reliability of SNNs. Low device‐to‐device and cycle‐to‐cycle variations demonstrate the robust operation of the SNN with slight accuracy degradation. These results highlight the remarkable capabilities and potential of DG MPBTFT‐based LIF neurons, opening new frontiers for advanced neuromorphic computing systems.

## 4 Experimental Section

### Fabrication of DG MPBTFTs, FeTFTs, and Normal TFTs

The DG MPBTFTs, FeTFTs, and normal TFTs were fabricated on a 6‐inch Si wafer. The fabrication was conducted as follows: First, a buffer oxide (SiO_2_, 300 nm) was grown on the Si substrate. Next, a 40 nm Mo layer was deposited as the BG of MPBTFTs and FeTFTs via direct current (DC) sputtering and patterned using a dry‐etching process (Figure S1a, Supporting Information). An 8 nm HZO (Hf:Zr = 2:1) layer was deposited as a nonvolatile ferroelectric layer of FeTFTs via thermal atomic layer deposition (ALD) (Figure S1b, Supporting Information). Subsequently, a 20 nm TiN layer was deposited via plasma‐enhanced ALD (PEALD) and patterned for an inner metal (IM) layer of FeTFTs (Figure S1b, Supporting Information). A nonvolatile HZO thin film was isolated through a wet‐etching process using a dilute HF (DHF) solution (Figure S1c, Supporting Information). Subsequently, a 6.5 nm HZO (Hf:Zr = 3:5) layer near the MPB was deposited as a volatile thin film of MPBTFTs via thermal ALD (Figure S1d, Supporting Information). Further, a 20 nm Mo layer was deposited via DC sputtering and patterned for both the IM layer of MPBTFTs and the BG of normal TFTs (Figure S1e, Supporting Information). A volatile HZO thin film near the MPB was isolated via a wet‐etching process using a DHF solution (Figure S1f, Supporting Information). Subsequently, rapid thermal annealing (RTA) was performed at 500 °C under N_2_ ambient conditions to induce crystallinity in the deposited thin films. MFM structures with different characteristics for nonvolatile FeTFTs and volatile MPBTFTs were formed via these processes. After depositing an 8 nm ZrO_2_ layer via thermal ALD as the insulator layer (Figure S1g, Supporting Information), a 30 nm amorphous IGZO channel was deposited via RF sputtering and wet‐etched using a diluted HCl solution (Figure S1h, Supporting Information). Then, Ti and Al were deposited via DC sputtering and patterned as the source (S) and drain (D) using a dry‐etching process (Figure S1i, Supporting Information). The annealing process was performed at 350  °C for 1 h under O_2_ ambient conditions to induce conduction in IGZO channels by forming oxygen vacancies. Some of these oxygen vacancies migrated to the interface between IGZO and the S/D metal, effectively reducing contact resistance. Finally, an 8 nm ZrO_2_ layer was deposited via thermal ALD (Figure S1j, Supporting Information), followed by a Mo deposition via DC sputtering and patterned for TG of the MPBTFTs using a dry‐etching process (Figure S1k, Supporting Information). The DG MPBTFTs and FeTFTs have IGZO channels with a width (*W*) and length (*L*) of 20 µm. The normal TFTs have a *W* and *L* of 40 µm and 20 µm, respectively.

### Electrical Measurements

A probe station and semiconductor parameter analyzer (Keithley 4200‐SCS) were employed to investigate the electrical characteristics of the fabricated DG MPBTFTs, FeTFTs, and normal TFTs. The DC *I*_D_–*V*_GS_ measurements were performed using a semiconductor parameter analyzer (Keysight B1500A). In addition, B1500A, equipped with a waveform generator/fast measurement unit (WGFMU) module, was utilized for pulse measurements. A probe station, custom‐made probe card, semiconductor parameter analyzer (Keysight 4156B), and pulse generator (Keysight 81110A) were used to assess the electrical characteristics of devices within the array. Inputs from each source were distributed across the probe card using a switching matrix (Keysight E5250A). All electrical measurements and characterizations were performed in ambient air at room temperature.

### MNIST Dataset Recognition

A 784 × 200 all‐ferroelectric fully connected single‐layer SNN was used for the MNIST dataset recognition task. The original 28 × 28 pixel grayscale images from the MNIST dataset were converted into binary images. Each pixel in the input image was encoded into an input pulse train using a rate coding method (Figure S15, Supporting Information). Therefore, the number of pulses in each input pulse train is proportional to the pixel intensity. These encoded input pulse trains are then fed into the input layer of the SNN, where the synaptic FeTFTs transmit signals to the DG MPBTFT‐based LIF neurons. Notably, the previously necessary synaptic device pairs for representing negative weights are eliminated because synaptic weights only have positive values, thereby enhancing area and energy efficiency.^[^ ^2^, ^5^, ^69^, ^70^ ^]^ When a LIF neuron exceeds a certain threshold, it fires, transmitting the signal to the subsequent layer and resetting the neuron to its initial state. For the SFA function, the *V*_TG_ applied to the LIF neuron is decreased with each neuronal firing. This effectively modulates the firing frequency of LIF neurons. Although a single‐layer SNN was utilized in this study, larger SNNs with hidden layers could be utilized (see Figure S13 (Supporting Information) for classification accuracy across various network sizes). Simulations were conducted using the measured electrical characteristics of synaptic FeTFTs, DG MPBTFT‐based LIF neurons, and normal IGZO TFTs.

### Synaptic Weight Update

The spike‐timing‐dependent plasticity (STDP) learning rule, which emulates the weight changes in biological synapses, was used for synaptic weight updates (refer to Figure S16 (Supporting Information) for details on the STDP learning rule). The synaptic weights were initialized based on the initialization method proposed by K. He.^[^ ^71^ ^]^ Following the STDP learning rule, synaptic weights are updated based on the time difference between the presynaptic and postsynaptic spikes connected to the synapse. The synaptic weight decreases when an output neuron is fired without a corresponding presynaptic spike at the connected synaptic device, which reflects long‐term depression (LTD). In contrast, if an output neuron is fired in conjunction with a presynaptic spike at the connected synaptic device, the synaptic weight increases, implying long‐term potentiation (LTP). Typically, synaptic devices exhibit nonlinear weight updates expressed by the following equations^[^ ^6^, ^66^ ^]^:

(1) $$G\left(n+1\right)=G\left(n\right)+\alpha_{p}exp\left(-\beta_{p}\frac{G\left(n\right)-G_{min}}{G_{max}-G_{min}}\right)$$

(2) $$G\left(n+1\right)=G\left(n\right)-\alpha_{d}exp\left(-\beta_{d}\frac{G_{max}-G\left(n\right)}{G_{max}-G_{min}}\right)$$

where *G*(*n*) represents the conductance of the synaptic device after *n* pulses; *G*_max_ and *G*_min_ represent the maximum and minimum conductance, respectively; and *α*_p_ and *β*_p_ represent the fitting parameters for the LTP characteristics, while *α*_d_ and *β*_d_ represent the fitting parameters for the LTD characteristics. Parameters *β*_p_ and *β*_d_ determine the nonlinearity in LTP and LTD characteristics. The fabricated synaptic FeTFTs demonstrated high linearity in weight updates, as indicated by their very small *β*_p_ and *β*_d_ values, thereby ensuring the high performance of the SNN (fitting parameters for the LTP and LTD characteristics of the synaptic FeTFTs are provided in Table S1, Supporting Information). Although this study employed the bio‐plausible STDP learning rule, the proposed DG MPBTFT‐based LIF neurons can be utilized with the backpropagation algorithm, potentially yielding higher classification accuracy. This is because the behavior of LIF neurons in SNNs can approximate the rectified linear unit (ReLU) activation function used in conventional deep neural networks.^[^ ^66^ ^]^

### Device Variations

In hardware‐based SNNs, the device‐to‐device variations are modeled for network simulations using the following equation:

(3) $$\Delta G_{measured}=\Delta G_{ideal}\times N\left(1,\sigma^{2}\right)$$

where ∆*G*_measured_ and ∆*G*_ideal_ represent the measured and ideal conductance changes in the device, respectively, and *N*(1, *σ* ^2^) represents a normal distribution with a mean of 1 and variance *σ* ^2^. The measured device variations of the synaptic FeTFTs and DG MPBTFT‐based LIF neurons were used in the simulations.

### Code Availability

All relevant codes used for the simulation are available within the article and its Supplementary Information or from the corresponding authors upon reasonable request.

## Conflict of Interest

The authors declare no conflict of interest.

## Supporting information



Supporting Information

## Data Availability

The data that support the findings of this study are available in the supplementary material of this article.
