# Supplementary material for: All‐Ferroelectric Spiking Neural Networks via Morphotropic Phase Boundary Neurons
Source: Adv Sci (Weinh). 2024 Oct 9;11(44):2407870. doi: 10.1002/advs.202407870 (PMC11892495; doi:10.1002/advs.202407870)
Supplement: Supplementary file 1 — Supporting Information [file ADVS-11-2407870-s001.docx]

Supplementary Information

**All-Ferroelectric Spiking Neural Networks**

**via Morphotropic Phase Boundary Neurons**

*Jangsaeng Kim^1,2†^, Eun Chan Park^1†^, Wonjun Shin^3†^, Ryun-Han Koo^2†^, Jiseong Im^2^, Chang-Hyeon Han^1^, Jong-Ho Lee^2,4^, and Daewoong Kwon^1^**

^1^Department of Electronic Engineering, Hanyang University, Seoul 04763, Republic of Korea

^2^Department of Electrical and Computer Engineering and Inter-university Semiconductor Research Center, Seoul National University, Seoul 08826, Republic of Korea

^3^Department of Semiconductor Convergence Engineering, Sungkyunkwan University, Suwon 16419, Republic of Korea

^4^Ministry of Science and ICT, Sejong 30121, Republic of Korea

*^†^ These authors contributed equally: Jangsaeng Kim, Eun Chan Park, Wonjun Shin, Ryun-Han Koo*

** Corresponding author (e-mail: dw79kwon@hanyang.ac.kr)*

**Contents**

Supplementary Figure S1–S16

Supplementary Table S1

Supplementary Note S1–S3

Supplementary References

**Supplementary Figures**


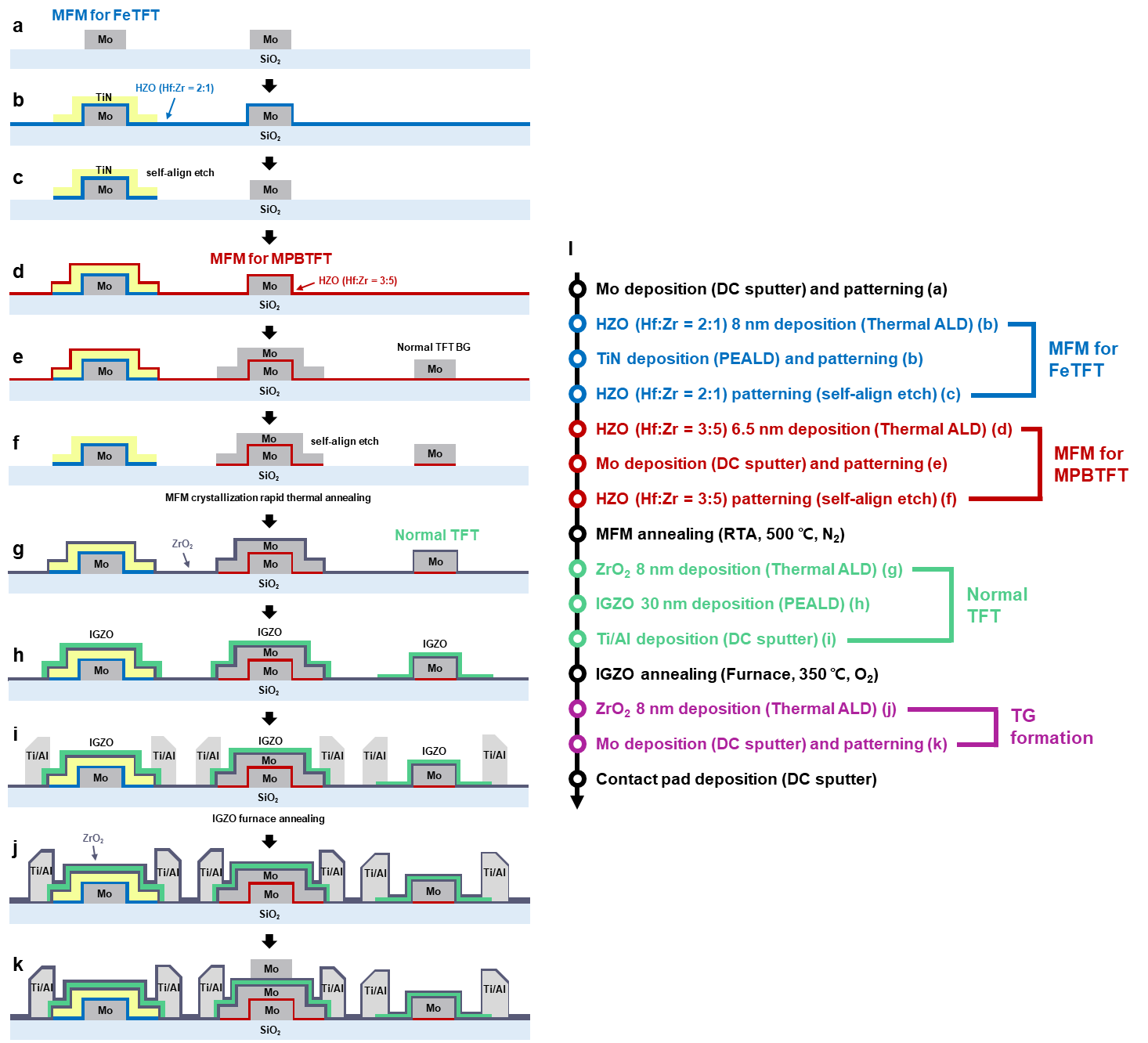


**Supplementary Figure S1.** **Fabrication processes for DG MPBTFTs, FeTFTs, and normal TFTs.** **a**–**k** Schematic views of key process steps. **l** Key process steps for co-integrating DG MPBTFTs, FeTFTs, and normal TFTs. The seamless co-integration of these TFTs on a single wafer facilitates the implementation of complete all-ferroelectric SNNs with enhanced area and process efficiencies.


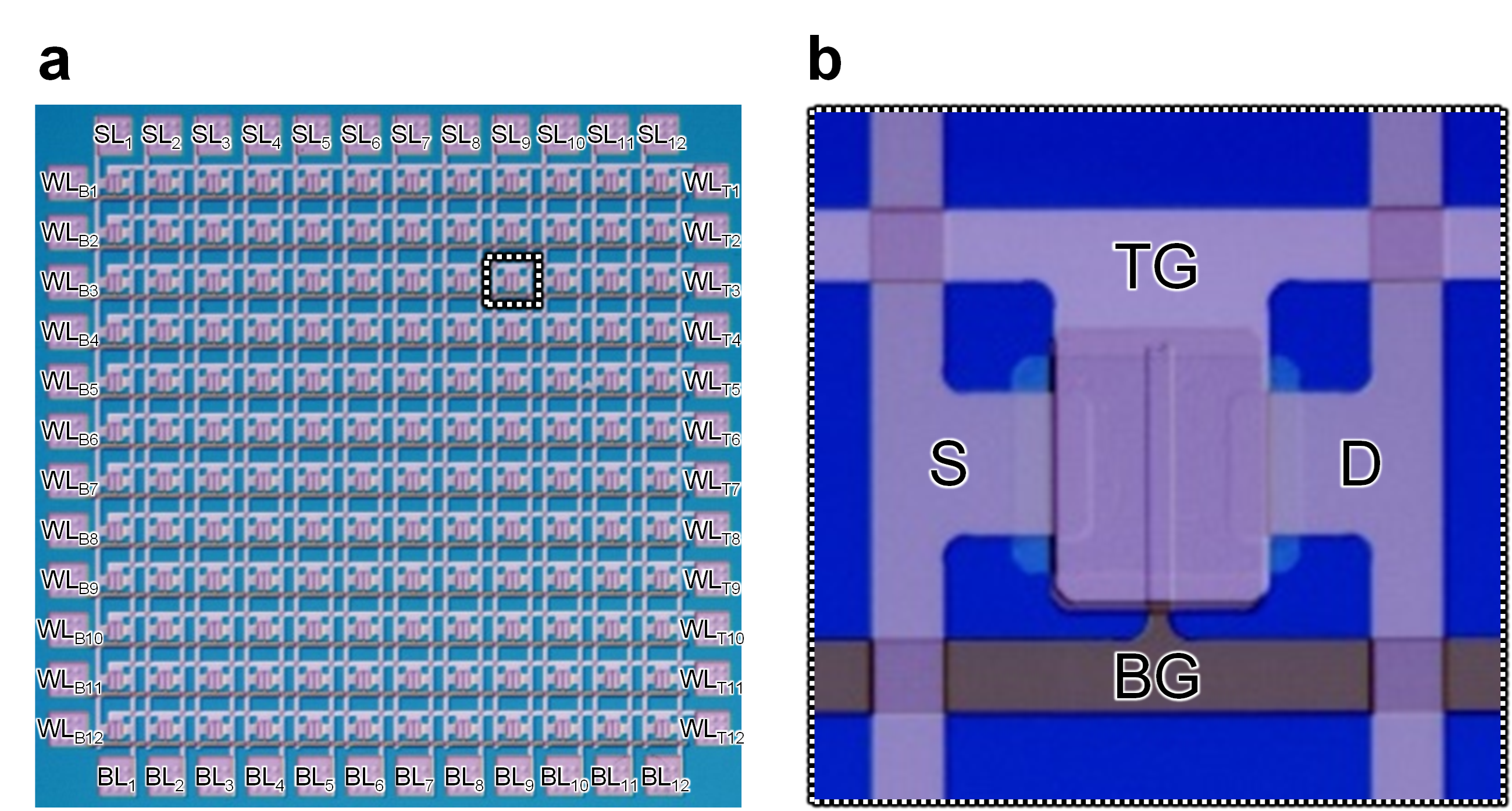


**Supplementary Figure S2.** **Top optical images of DG MPBTFT array. a** Top optical image of DG MPBTFT array, featuring word-line (WL) pairs (WL_B_ and WL_T_) arranged parallel to each other. **b** Magnified optical image of the white dashed line in (**a**).


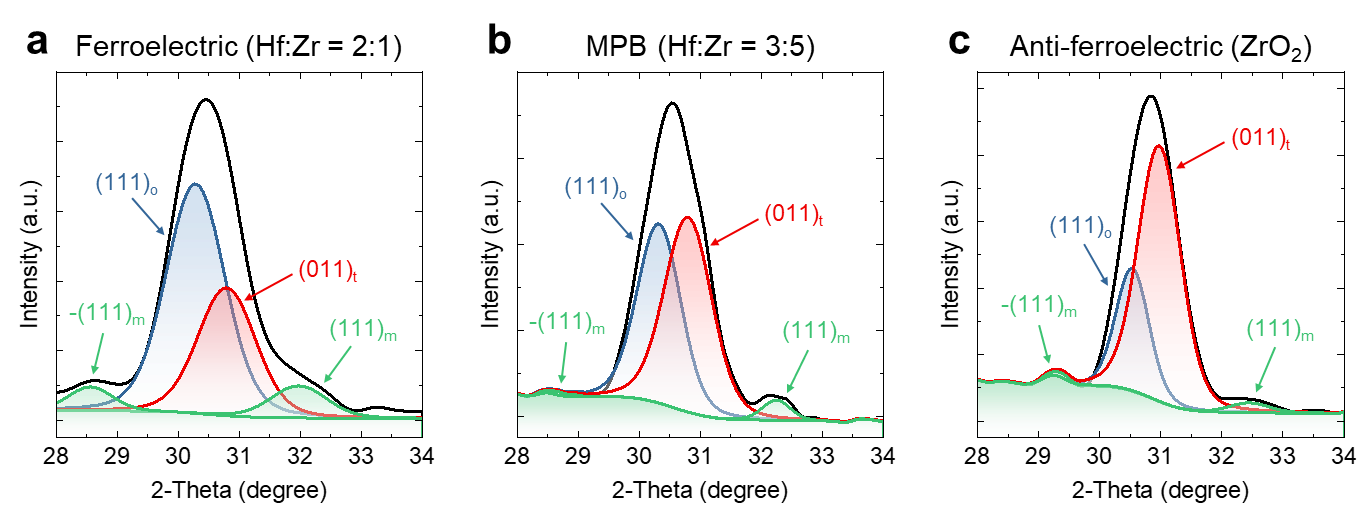


**Supplementary Figure S3.** **XRD analyses of HZO thin films with various Zr contents.** Deconvoluted XRD spectra of **a** ferroelectric, **b** MPB, and **c** anti-ferroelectric materials through the fixed peak positions. As Zr content in HZO thin film increases, the phase in HZO transit from the o-phase to the t-phase. The ferroelectric material primarily exhibits the o-phase, whereas the t-phase characterizes the anti-ferroelectric material. The MPB represents an intermediate state between these two phases. The relative phase ratios derived from XRD for each material type are shown in Figure 3h.


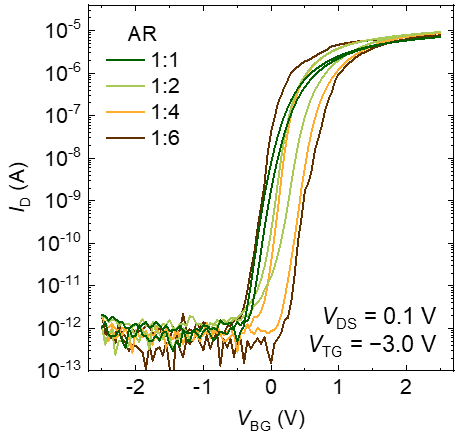


**Supplementary Figure S4.** **Hysteretic transfer characteristics of DG MPBTFT according to various area ratios (ARs).** The memory window increases with a decrease in AR. This is because an intensified electric field across the MFM layer enhances polarization within the MFM layer.


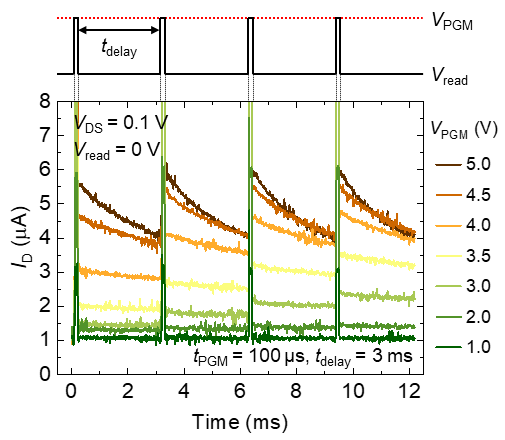


**Supplementary Figure S5.** ***I*_D_ response of DG MPBTFT to consecutive PGM pulses with various *V*_PGM_ conditions.** The upper panel illustrates the series of applied PGM pulses, which are followed by a constant read voltage. The time interval between each PGM pulse is fixed at 3 ms.


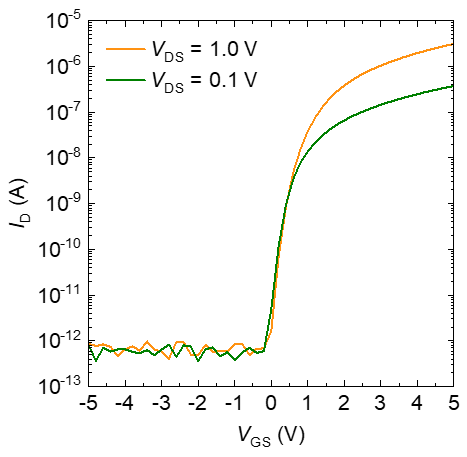


**Supplementary Figure S6. Transfer characteristics of normal IGZO TFT.** The fabricated normal IGZO TFTs demonstrate a high on/off current ratio with sub-pA off current. The employment of IGZO as a channel material presents several advantages, including the ability to fabricate at temperatures below the threshold required to induce ferroelectricity in HZO films [1]. This characteristic enables incorporating IGZO channels into ferroelectric devices while preserving the ferroelectric properties of HZO films.


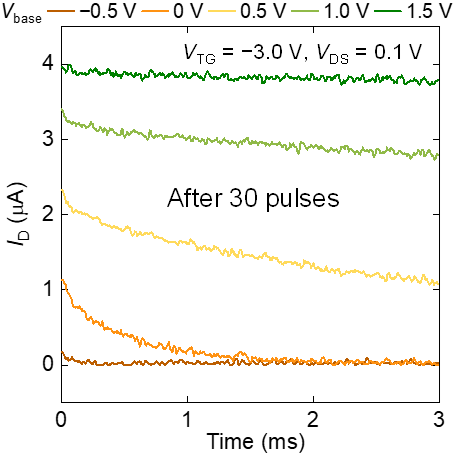


**Supplementary Figure S7.** **Spontaneous reset characteristics of DG MPBTFT-based LIF neurons under various *V*_base_ conditions.** Identical 30 input pulses with *V*_high_, *t*_width_, and *t*_delay_ of 2.5 V, 500 μs, and 10 μs, respectively, are applied.


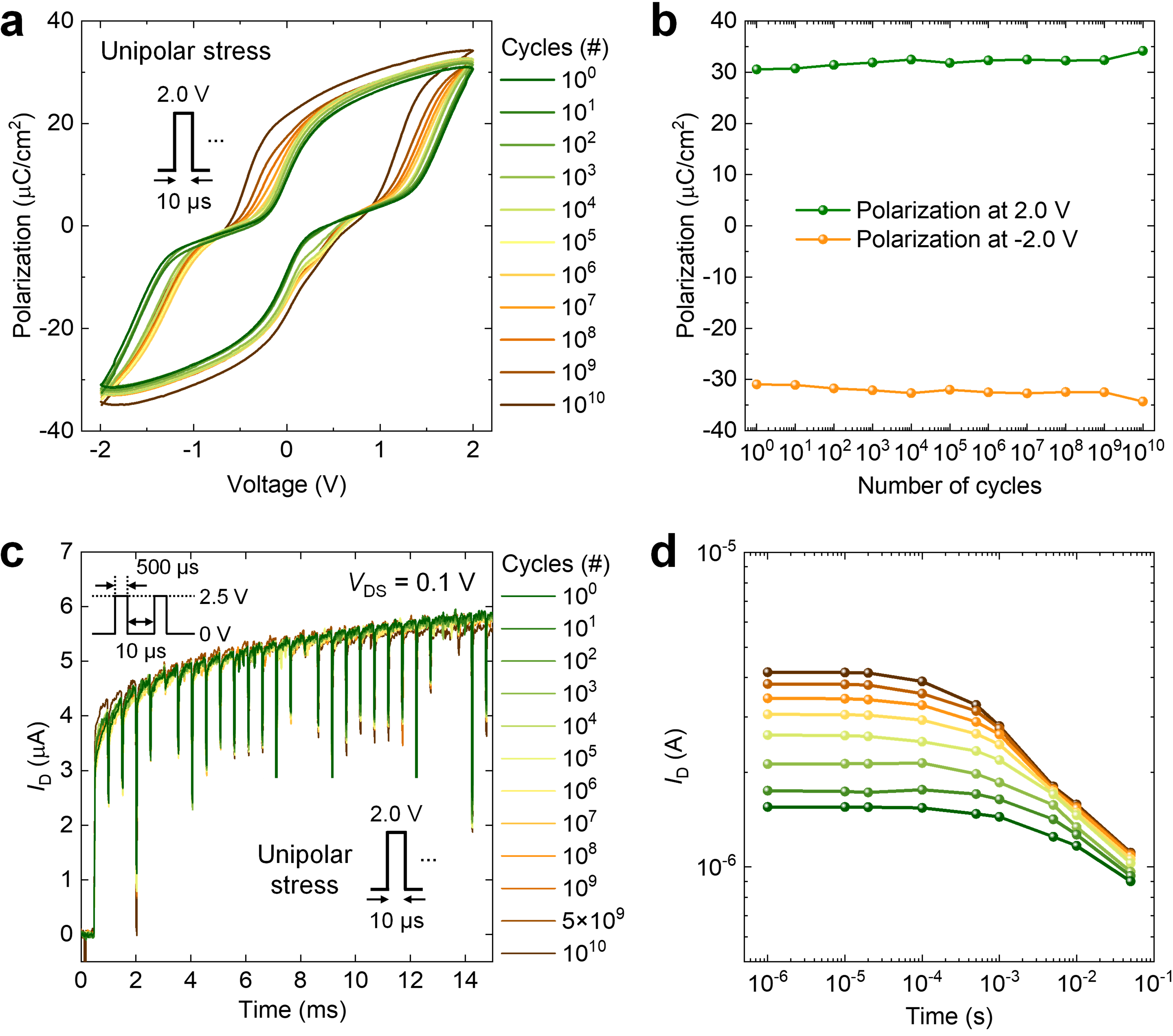


**Supplementary Figure S8.** **Endurance and retention characteristics of DG MPBTFT-based LIF neurons. a** MFM structure with MPB layer exhibits high endurance of more than 10^10^ cycles under unipolar stress. **b** Polarization of the MFM structure with MPB layer for the number of cycles. **c** Stable neuronal operation of DG MPBTFT-based LIF neurons across 10^10^ firing cycles. The neurons demonstrate significant robustness for numerous firing cycles. The endurance measurements were conducted for unipolar stress because input pulses with only positive voltage are used for neuronal operation. Ferroelectric devices featuring an MFMIS structure are renowned for their robust endurance characteristics [2, 3]. **d** Retention characteristics of DG MPBTFT at room temperature. The device exhibits volatile memory characteristics.


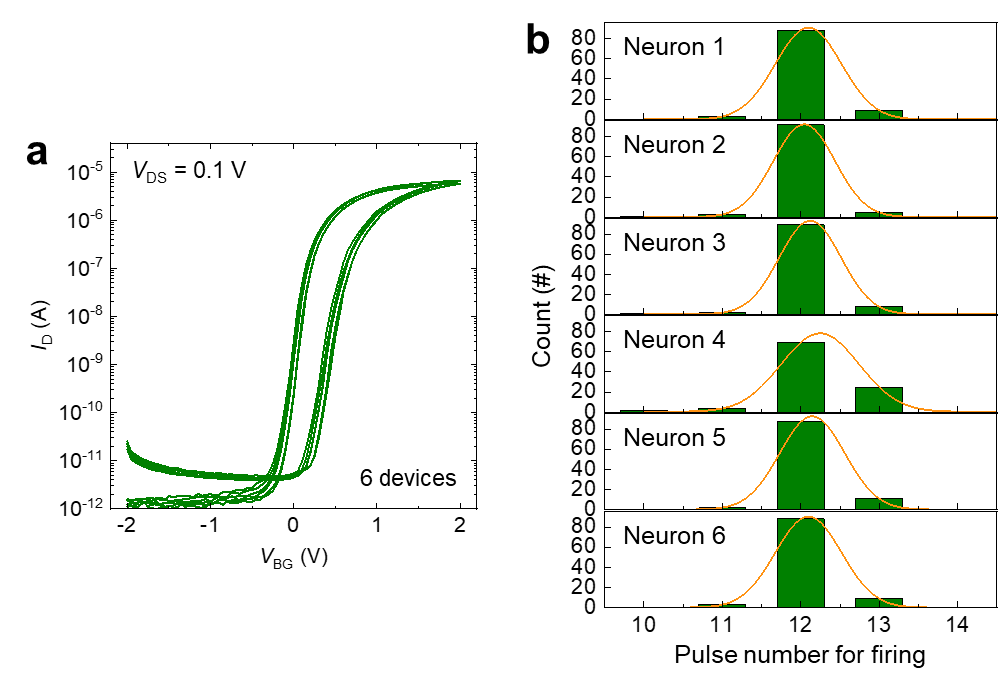


**Supplementary Figure S9.** **Device-to-device variation of DG MPBTFTs. a** Hysteretic transfer characteristics of six DG MPBTFTs exhibiting a low device-to-device variation of 7.51%. **b** Histograms of the number of pulses required to trigger neuronal firing for six DG MPBTFT-based LIF neurons. Across 600 neuronal firing activities, the neurons demonstrate high uniformity in neuronal operations.


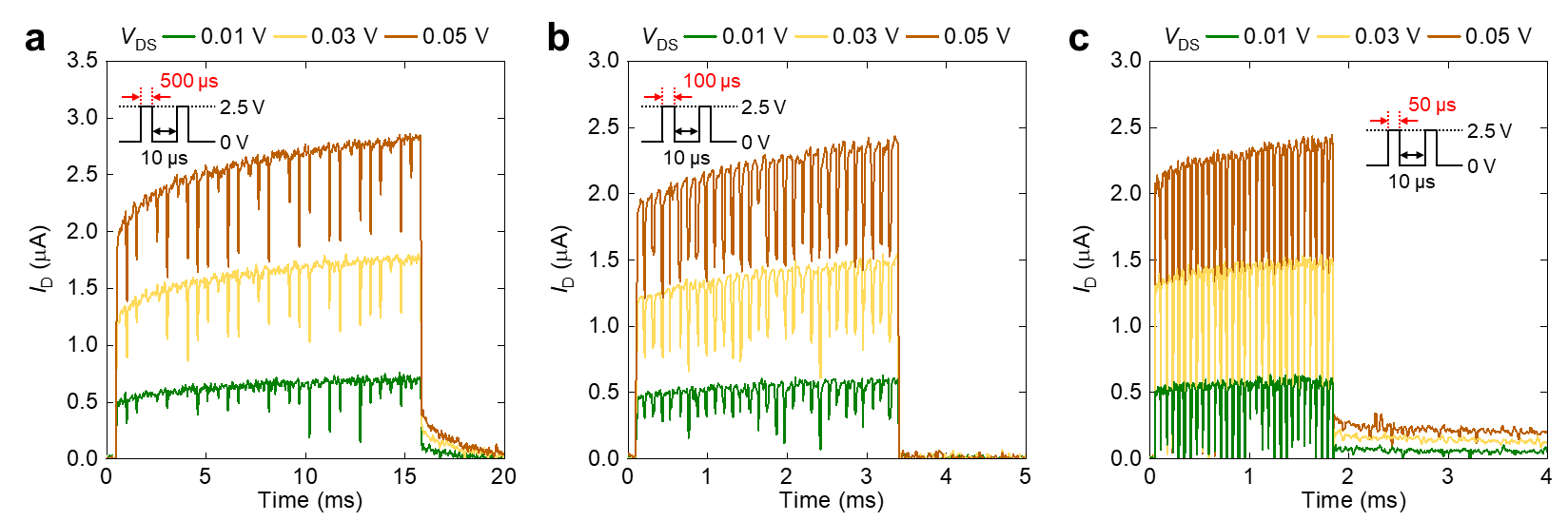


**Supplementary Figure S10.** **Neuronal behavior of DG MPBTFTs with various *V*_DS_ and *t*_width_ conditions.** Neuronal behavior under *t*_width_ of **a** 500 μs, **b** 100 μs, and **c** 50 μs with various *V*_DS_ conditions. The insets show the input pulse train conditions. The *I*_D_ of the DG MPBTFTs decreases with a decrease in *V*_DS_ and *t*_width_, while maintaining the integration and reset functions. Stable neuronal operations are verified, offering the possibility of further minimizing the energy consumption of the neurons.


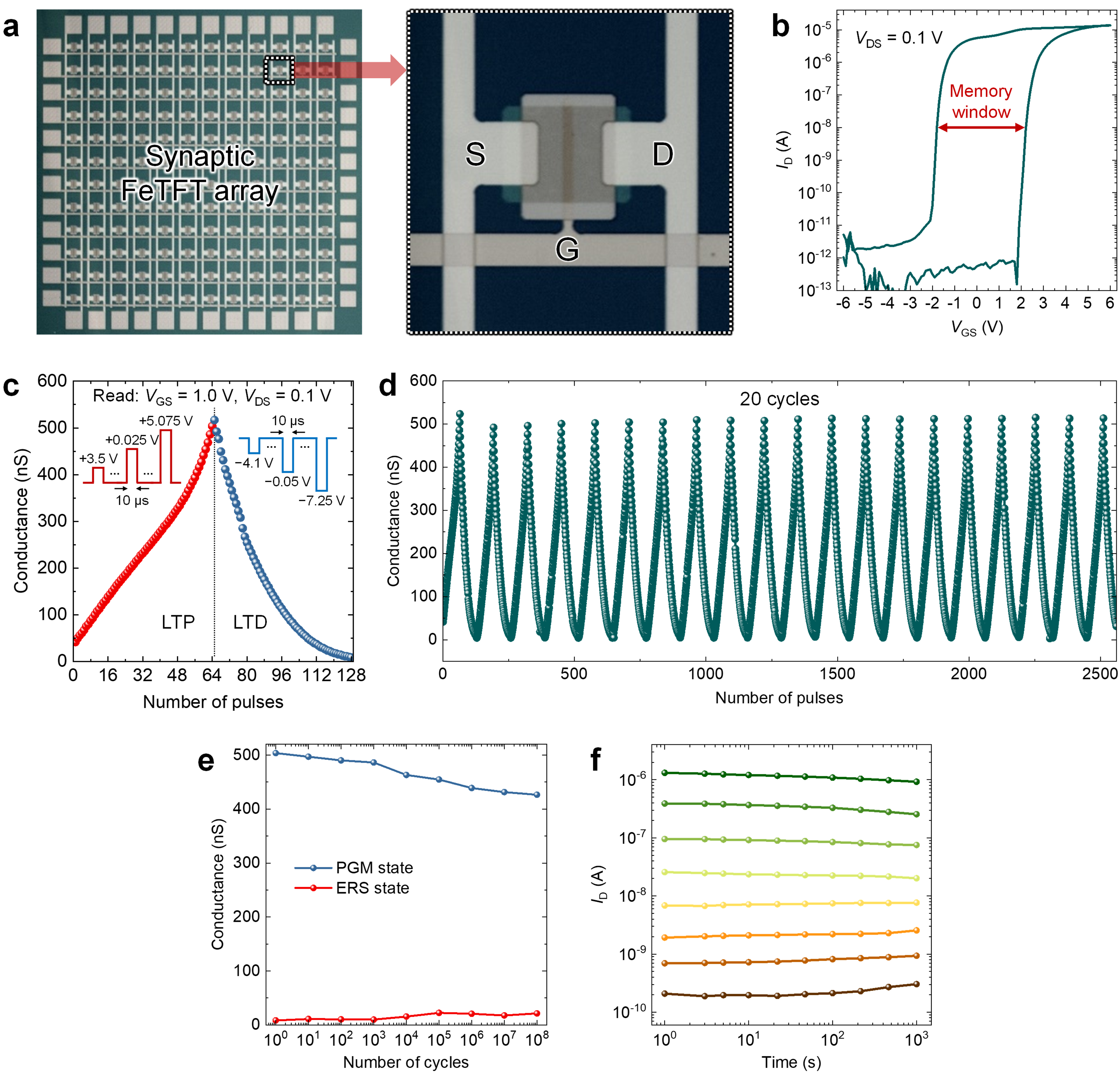


**Supplementary Figure S11.** **Electrical characteristics of synaptic FeTFTs. a** Top optical images of synaptic FeTFT array. A magnified optical image of the white dashed line is presented. **b** Hysteretic transfer characteristic of the FeTFTs with wide memory window of approximately 4 V at a constant *I*_D_ of 10 nA. **c** Long-term potentiation (LTP) and long-term depression (LTD) characteristics of the FeTFTs with the number of applied pulses. The insets show the schematics of applied PGM and ERS pulses. The synaptic FeTFTs demonstrate multilevel synaptic weights (6 bits) with highly linear weight update characteristics. **d** LTP and LTD characteristics over 20 cycles, with each cycle containing 128 applied pulses. The synaptic FeTFTs exhibit a low cycle-to-cycle variation of 9.19%, ensuring high reliability. **e** Endurance and **f** Retention characteristics of the FeTFTs at room temperature.


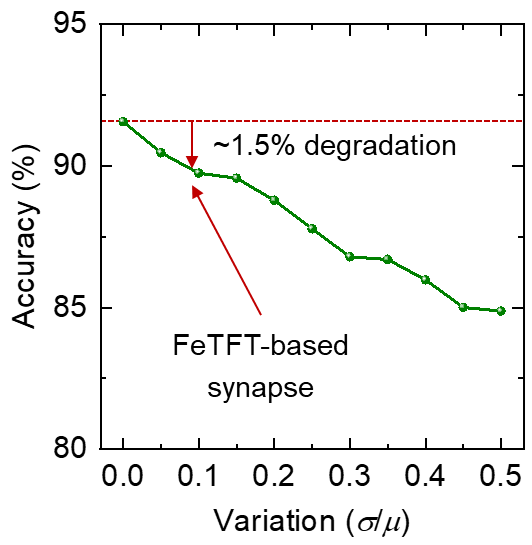


**Supplementary Figure S12.** **Classification accuracy of SNN as a function of the cycle-to-cycle variation of FeTFT-based synapses.** The SNN using synaptic FeTFTs demonstrates stable operation with a slight accuracy degradation of about 1.5% owing to a low cycle-to-cycle variation.


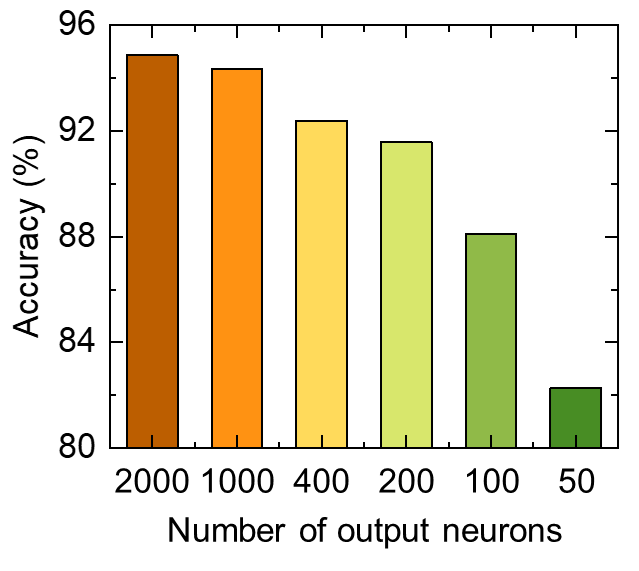


**Supplementary Figure S13.** **Classification accuracy of SNNs for various network sizes.** The performance of the SNN is enhanced with an increase in the number of output neurons.


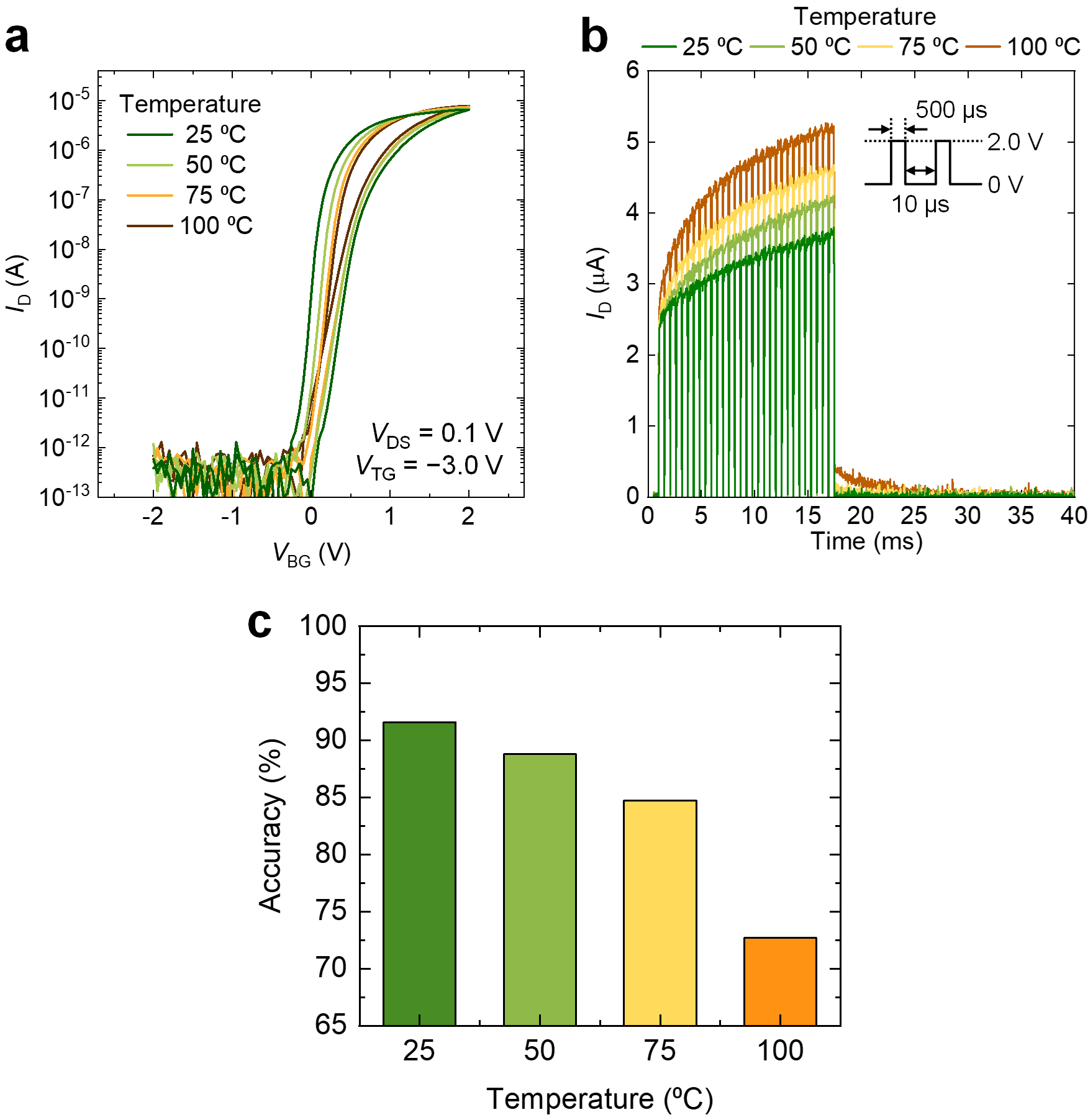


**Supplementary Figure S14.** **Impacts of temperature on neuronal behavior and SNN performance. a** Hysteretic transfer characteristics of DG MPBTFT according to various temperatures. **b** Neuronal behavior of DG MPBTFTs for various temperatures. **c** Classification accuracy of SNNs for various temperatures. The number of output neurons is 200.


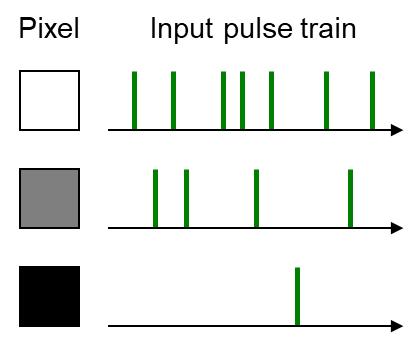


**Supplementary Figure S15.** **Rate coding method for input image encoding.** The number of input pulses in each pulse train is proportional to the pixel intensity of the input image. Although this study employed the rate coding method, other input encoding methods, such as temporal coding, phase coding, burst coding, and time-to-first-spike coding, can also be used [4].


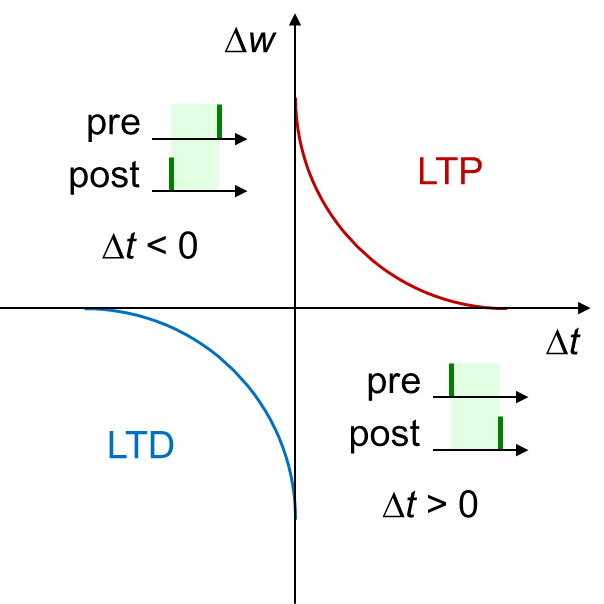


**Supplementary Figure S16. Spike-timing-dependent plasticity (STDP) learning rule.** ∆*w* and ∆*t* represent a change in synaptic weight and the time difference between presynaptic and postsynaptic spikes, respectively. The synaptic weights are updated according to the time difference between presynaptic and postsynaptic spikes. The synaptic weights increase when presynaptic spikes are applied before the postsynaptic spikes, and vice versa. Modified learning rules, such as simplified STDP, can also be used for synaptic weight updates to minimize hardware burden [5].

**Supplementary Tables**


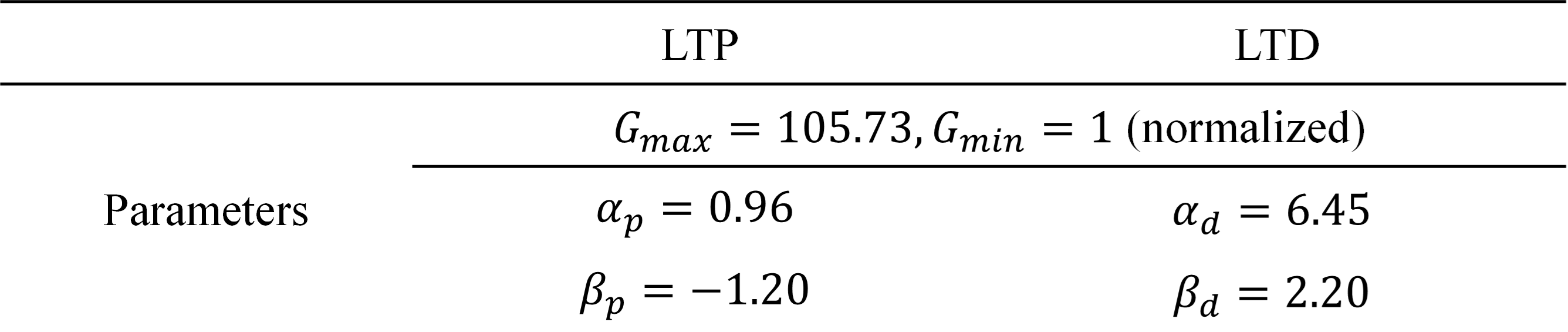


**Supplementary Table S1. Nonlinearity fitting parameters for LTP and LTD characteristics of synaptic FeTFTs.** The fabricated synaptic FeTFTs demonstrate a highly linear conductance response, which contributes to the high performance of the SNN. The nonlinearity of the synaptic FeTFTs can be further minimized by optimizing the PGM and ERS pulse schemes.

**Supplementary Notes**

**Supplementary Note S1. Advantages of our DG MPBTFT-based LIF neurons**

In the realm of neuromorphic computing, the fundamental challenge of implementing artificial neurons lies in addressing specific needs such as low power consumption, high endurance, elimination of membrane capacitors and reset circuits, and capability to implement spike-frequency adaptation (SFA) and lateral inhibition functions. Despite the exploration of various emerging devices to meet these requirements, achieving all simultaneously has remained a formidable challenge (Table 1). While many studies have focused on individual neuron functionalities, only a limited number have delved into the integration with artificial synapses. To address these challenges, we propose area- and energy-efficient artificial neurons with enhanced functional versatility through the co-optimization of material properties and device configurations. Our artificial neurons have the following advantages:

1) We report, for the first time, artificial leaky integrate-and-fire (LIF) neurons by leveraging morphotropic phase boundary (MPB), which was previously limited to use as a cell capacitor in dynamic random-access memory (DRAM). The systematic engineering of the material versatility of hafnium zirconium oxide (HZO) leads to the implementation of area- and energy-efficient LIF neurons. The engineered HZO films near the MPB exhibit a high polarization at relatively lower voltages than anti-ferroelectric materials (Figures 3e and f), facilitating low-power neuronal operations. Inherent partial polarization switching with spontaneous reset characteristics demonstrates integration and reset functions of biological neurons without the need for capacitors and complex reset circuits (Figure 4b), while maintaining high endurance (Supplementary Figure S8). This study provides the potential of MPB-based thin-film transistors (MPBTFTs) as artificial neurons, fulfilling the crucial criteria essential for efficient neuromorphic computing.

2) In addition to material engineering, the double-gate (DG) configuration of MPBTFTs enhances the functional versatility of the LIF neurons. The DG MPBTFTs with four terminals effectively implement SFA and lateral inhibition functions, which are critical neural mechanisms (Figure 6). The ferroelectric mechanism described in the manuscript affects polarization switching through both the bottom-gate (BG) and the top-gate (TG). Therefore, the degree of polarization switching by input pulses can be modulated by varying the bias applied to the TG while input pulses are applied to the BG. This capability was utilized to implement the SFA function in this work. The SFA function leveraging the DG configuration effectively modulates the neuronal firing frequency (Figure 6c), significantly enhancing the performance of spiking neural networks (SNNs) (Figure 7d). The DG MPBTFT-based LIF neurons with high CMOS compatibility are seamlessly co-integrated with synaptic ferroelectric thin-film transistors (FeTFTs) on a single wafer, demonstrating all-ferroelectric SNNs (Supplementary Figure S1). Our fully integrated SNN using the DG MPBTFT-based LIF neurons achieved a high classification accuracy of 94.9% on the MNIST dataset (Supplementary Figure S13). The consistent neuronal operations maintained across 10^10^ firing cycles (Supplementary Figure S8) with low device-to-device and cycle-to-cycle variations (Supplementary Figure S9) demonstrate excellent reliability and robustness.

With this point of view, we believe that our DG MPBTFT-based LIF neurons will open a new paradigm for researchers in the field of artificial neurons and novel applications of MPBTFTs, and provide insights to address the limitations of conventional artificial neurons.

**Supplementary Note S2. HZO thin films near the MPB**

The MPB between the ferroelectric and anti-ferroelectric states is utilized for artificial neurons with high area and energy efficiencies. The crystallization of HfO_2_–ZrO_2_ mixed thin films primarily produces the o-phase and t-phase, foundational to the ferroelectric properties observed in HfO_2_-based materials [6–8]. Various factors, including material composition, grain size, thickness of films, and external stress, influence the phase evolution in HZO thin films. In this study, the MPB is obtained through precise modulation of the composition ratio of ZrO_2_ in HZO thin films, which is the paramount factor affecting the polarization switching dynamics within the films.

Several metastable crystalline phases emerge in the HZO thin films near the MPB. The asymmetric interstitial distance of ZrO_2_ greater than that of HfO_2_ facilitates a phase transition from the o-phase to the t-phase with an increase in ZrO_2_ content, reducing the total energy of the grain. A high ZrO_2_ content leads to a reduction in coercive voltage and diminishes the energy barrier between the two phases, thereby flattening the mixed phase between the paraelectric and ferroelectric phases. This phase is characterized by the absence of hysteresis and a high dielectric constant [9–13]. Park et al. suggested that the enhanced dielectric constant might be due to reversible phase boundary motion between the o-phase (ferroelectric) and t-phase (anti-ferroelectric) [14]. Films with a higher Zr concentration predominantly exhibit the t-phase, which is associated with increased permittivity. The high dielectric constant observed in the HZO near the MPB can also be attributed to the smaller grain size of the films dominated by the t-phase compared to other phases [15–17]. The relationship between grain size and dielectric constant is well-established, where smaller grain sizes typically correspond to higher dielectric constants [18, 19]. While the HZO thin films near the MPB have been primarily employed in DRAM technology owing to their high dielectric constant, we focused on their potential for low-power operation and spontaneous reset functions in artificial neurons.

XRD analyses were conducted for HZO with various Zr contents (Supplementary Figure S3). While the stable m-phase (111)_m_ is identified by its distinct diffraction peaks, the o-phase (111)_o_ and t-phase (011)_t_ exhibit similar peaks. The relative phase fractions for the (111)_o_ and (011)_t_ were calculated based on the assumption that their respective diffraction peaks occur at 30.4° and 30.8° [15, 20, 21].

While the complex interplay between ferroelectric polarization and the migration of internal oxygen vacancies remains to be fully elucidated, existing research has established that vacancy migration is closely linked with ferroelectric switching in HZO thin films [22–24]. It has been reported that the presence of oxygen vacancies in the HZO can reduce energy barriers, thereby assisting polarization switching. Notably, the formation of oxygen vacancies can be managed by incorporating an oxygen scavenging layer between the HZO layer and the electrode [25, 26]. This oxygen scavenging layer facilitates the generation of positively charged oxygen vacancies at the interface between the HZO and oxygen scavenging layer via an oxidation-reduction process.

Supplementary Note Figure 1 shows the *P*–*V* hysteresis loop for MPB materials with various voltage amplitudes and frequencies.


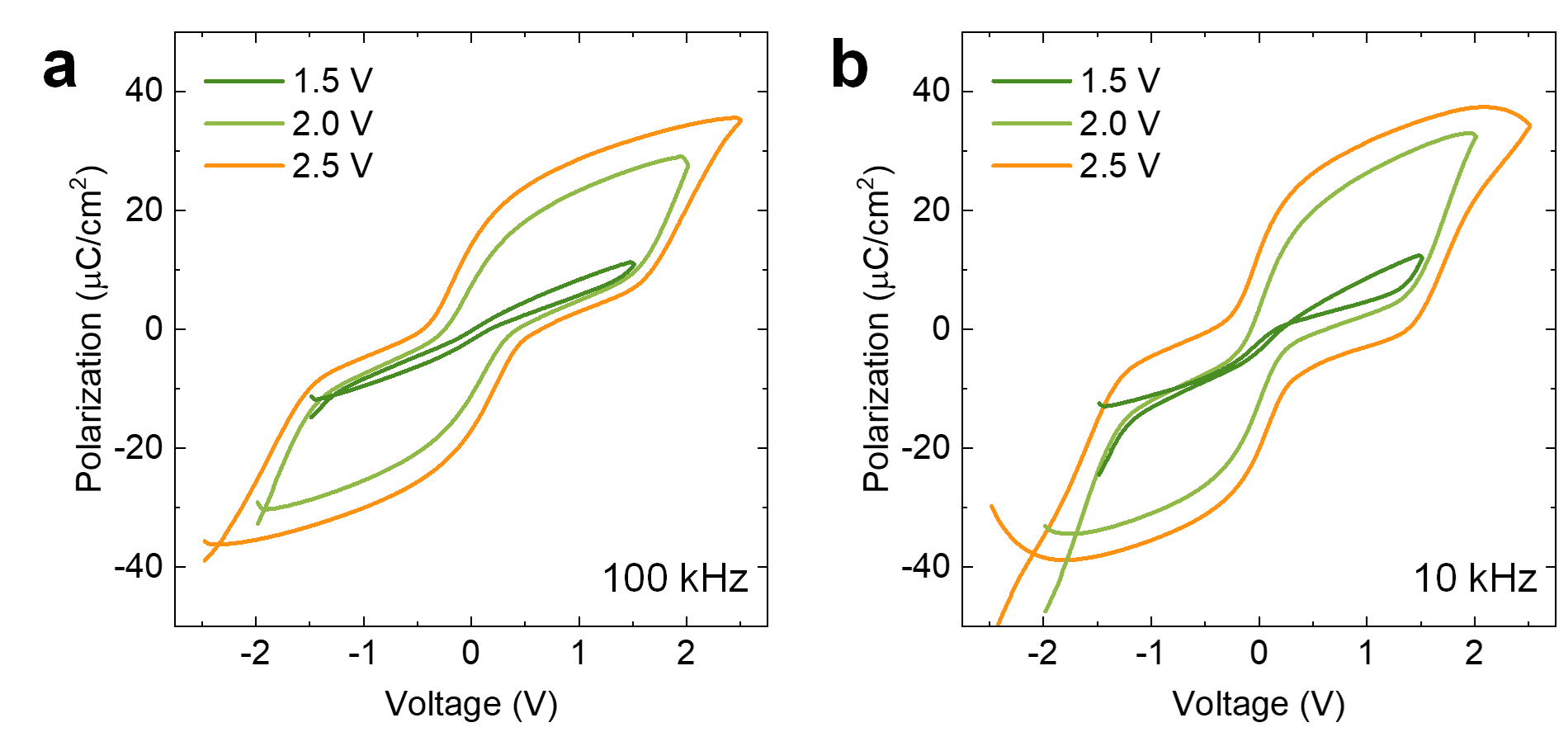


**Supplementary Note Figure 1.** *P*–*V* hysteresis loop for MPB materials with various voltage amplitudes measured at **a** 100 kHz and **b** 10 kHz.

**Supplementary Note S3. Estimation of energy consumption**

The energy consumption of the DG MPBTFT-based LIF neurons ($E_{neuron}$) is obtained from the neuronal operation in Supplementary Figure S10c and is quantitatively estimated as follows,

$E_{neuron}=V_{neuron}\times I_{neuron}\times t_{width}=0.03 V\times1.3 \mu A\times50 \mu s=1.95 pJ/spike$, (1)

where $V_{neuron}$ is the read voltage for the neuron, $I_{neuron}$ is the average current response of the DG MPBTFTs, and $t_{width}$ is the pulse width. We assume $I_{neuron}$ of 1.3 μA from the current response characteristics of the DG MPBTFT (ranges from ~1.1 μA to ~1.5 μA). Such energy consumption is significantly lower than that of the state-of-the-art artificial neurons using emerging devices (Table 1).

The energy consumption of the FeTFT-based synapses ($E_{synapse}$) is quantitatively estimated as follows,

$E_{synapse}={V_{synapse}}^{2}\times G_{synapse}\times t_{width}={(0.1 V)}^{2}\times250 nS\times50 \mu s=125 fJ/spike$, (2)

where $V_{synapse}$ is the read voltage for the synapse, and $G_{synapse}$ is the average conductance of the synaptic FeTFTs. We assume $G_{synapse}$ of 250 nS from the LTP/LTD characteristics of the synaptic FeTFTs (ranges from ~0 nS to ~500 nS). The energy consumption of the FeTFT-based synapses is much lower than that of the DG MPBTFT-based LIF neurons. The estimation results demonstrate that the DG MPBTFT-based LIF neurons exhibit high energy efficiency. Note that the energy efficiency can be further enhanced by reducing the read voltage and pulse width within the range of stable neuronal operation.

**Supplementary References**

1. Nomura, K. et al. Room-temperature fabrication of transparent flexible thin-film transistors using amorphous oxide semiconductors. *Nature* **432**, 488–492 (2004).
2. Sun, C. et al. First demonstration of BEOL-compatible ferroelectric TCAM featuring a-IGZO Fe-TFTs with large memory window of 2.9 V, scaled channel length of 40 nm, and high endurance of 10 8 cycles. *2021 Symposium on VLSI Technology*. IEEE (2021).
3. Wang, X. et al. First Demonstration of BEOL-Compatible MFMIS Fe-FETs with 3D Multi-Fin Floating Gate: In-situ ALD-deposited MFM, L CH of 50 nm,> 2× 10 9 Endurance, and 58.3% Area Saving. *2023 IEEE Symposium on VLSI Technology and Circuits (VLSI Technology and Circuits)*. IEEE (2023).
4. Xu, Q. et al. Hierarchical spiking-based model for efficient image classification with enhanced feature extraction and encoding. *IEEE Transactions on Neural Networks and Learning Systems* (2022).
5. Kim, J. et al. Demonstration of In-Memory Biosignal Analysis: Novel High-Density and Low-Power 3D Flash Memory Array for Arrhythmia Detection. *Adv. Sci.* 2308460 (2024).
6. Schroeder, U. et al. The fundamentals and applications of ferroelectric HfO2. *Nature Reviews Materials* **7**, 653–669 (2022).
7. Park, M. H. et al. Review and perspective on ferroelectric HfO2-based thin films for memory applications. *Mrs Communications* **8**, 795–808 (2018).
8. Mulaosmanovic, H. et al. Ferroelectric field-effect transistors based on HfO2: a review. *Nanotechnology* **32**, 502002 (2021).
9. Park, M. H. et al. Morphotropic Phase Boundary of Hf1–xZrxO2 Thin Films for Dynamic Random Access Memories. *ACS Applied Materials & Interfaces* **10**, 42666–42673 (2018).
10. Kashir, A. et al. Hf1–xZrxO2/ZrO2 Nanolaminate Thin Films as a High-κ Dielectric. *ACS Applied Electronic Materials* **3**, 5632–5640 (2021).
11. Müller, J. et al. Ferroelectricity in Simple Binary ZrO2 and HfO2. *Nano Letters* **12**, 4318–4323 (2012).
12. Chuang, C. H. et al. Sharp Transformation across Morphotropic Phase Boundary in Sub-6 nm Wake-Up-Free Ferroelectric Films by Atomic Layer Technology. *Adv. Sci.* **10**, 2302770 (2023).
13. Gaddam, V. et al. Novel Approach to High κ (~59) and Low EOT (~3.8 Å) near the Morphotrophic Phase Boundary with AFE/FE (ZrO2/HZO) Bilayer Heterostructures and High-Pressure Annealing. *ACS Appl. Mater. Interfaces* **14**, 43463–43473 (2022).
14. Park, J. Y. et al. Engineering Strategies in Emerging Fluorite-Structured Ferroelectrics. *ACS Applied Electronic Materials* **4**, 1369−1380 (2022).
15. Das, D., Gaddam, V. & Jeon, S. Demonstration of High Ferroelectricity (Pr ~ 29 μC/cm2) in Zr Rich HfxZr1–xO2 Films. *IEEE Electron Device Letters* **41**, 34–37 (2020).
16. Das, D., et al. Influence of High-Pressure Annealing Conditions on Ferroelectric and Interfacial Properties of Zr-Rich HfxZr1−xO2 Capacitors. *IEEE Trans. Electron Devices* **68**, 1996−2002 (2021).
17. Park, M. H. et al. Effect of Zr Content on the Wake-Up Effect in Hf1−xZrxO2 Films. *ACS Appl. Mater. Interfaces* **8**, 15466−15475 (2016).
18. Cai, Z. et al. Grain-Size−Dependent Dielectric Properties in Nanograin Ferroelectrics. *Journal of the American Ceramic Society* **101**, 5487-5496 (2018).
19. Gaddam, V. et al. Novel approach to high κ (∼ 59) and low EOT (∼ 3.8 Å) near the morphotrophic phase boundary with AFE/FE (ZrO2/HZO) bilayer heterostructures and high-pressure annealing. *ACS Applied Materials & Interfaces* **14**, 43463-43473 (2022).
20. Gaddam, V., Das, D. & Jeon, S. Insertion of HfO2 Seed/Dielectric Layer to the Ferroelectric HZO Films for Heightened Remanent Polarization in MFM Capacitors. *IEEE Transactions on Electron Devices* **67**, 745–750 (2020).
21. Park, M. H. et al. Evolution of phases and ferroelectric properties of thin Hf0.5Zr0.5O2 films according to the thickness and annealing temperature. *Appl. Phys. Lett.* **102**, 242905 (2013).
22. Nukala, P. et al. Reversible oxygen migration and phase transitions in hafnia-based ferroelectric devices. *Science* **372**, 630–635 (2021).
23. Kang, S. et al. Highly enhanced ferroelectricity in HfO2-based ferroelectric thin film by light ion bombardment. *Science* **376**, 731–738 (2022).
24. Noheda, B. et al. Lessons from hafnium dioxide-based ferroelectrics. *Nature Materials* **22**, 562–569 (2023).
25. Close, T. et al. Reversible oxygen scavenging at room temperature using electrochemically reduced titanium oxide nanotubes. *Nature Nanotechnology* **10**, 418–422 (2015).
26. Winter, R. et al. Effects of titanium layer oxygen scavenging on the high-k/InGaAs interface. *ACS Applied Materials & Interfaces* **8**, 16979–16984 (2016).
